# Experimental and Computational Insights into the Apoptotic Potential of New Phenanthroline-Based Copper(II) Complexes: From Spectroscopic Characterization and In Vitro Cytotoxicity to In Silico Target Identification

**DOI:** 10.3390/biomedicines14071625

**Published:** 2026-07-20

**Authors:** Jesús Magdiel García-Díaz, Héctor Alejandro Bacilio-Beltrán, Asbiel Felipe Garibaldi-Ríos, Martha Patricia Gallegos-Arreola, Irma Idalia Rangel-Salas, Jorge Iván Delgado-Saucedo, Paola Castro-García, Moisés Martínez-Velázquez, Ana María Puebla-Pérez

**Affiliations:** 1 Unidad de Biotecnología Médica y Farmacéutica, Centro de Investigación y Asistencia en Tecnología y Diseño del Estado de Jalisco A.C., Guadalajara 44270, Jalisco, Mexico; jegarcia_al@ciatej.edu.mx (J.M.G.-D.); mmartinez@ciatej.mx (M.M.-V.); 2 Departamento de Química, Centro Universitario de Ciencias Exactas e Ingenierías (CUCEI), Universidad de Guadalajara (UdeG), Guadalajara 44430, Jalisco, Mexico; alejandro.bacilio@alumnos.udg.mx (H.A.B.-B.); idalia.rangel@academicos.udg.mx (I.I.R.-S.); 3 División de Genética, Centro de Investigación Biomédica de Occidente (CIBO), Centro Médico Nacional de Occidente (CMNO), Instituto Mexicano Del Seguro Social (IMSS), Guadalajara 44340, Jalisco, Mexico; asbiel.garibaldi4757@alumnos.udg.mx (A.F.G.-R.); marthapatriciagallegos08@gmail.com (M.P.G.-A.); 4 Doctorado en Genética Humana, Centro Universitario de Ciencias de La Salud (CUCS), Universidad de Guadalajara (UdeG), Guadalajara 44340, Jalisco, Mexico; 5 Departamento de Farmacobiología, Centro Universitario de Ciencias Exactas e Ingenierías (CUCEI), Universidad de Guadalajara (UdeG), Guadalajara 44430, Jalisco, Mexico; jorge.delgado@academicos.udg.mx (J.I.D.-S.); paola.castro@academicos.udg.mx (P.C.-G.)

**Keywords:** copper(II) complexes, 1,10-phenanthroline, chemoinformatics, molecular docking, apoptosis, cytotoxicity

## Abstract

**Background**: Two novel copper(II) coordination complexes, PH-Cu [(1,10-phenanthroline)(malonato)copper(II)] and PC-Cu [(1,10-phenanthroline)(cyclobutane-1,1-dicarboxylato)copper(II)], were synthesized and evaluated as potential anticancer agents, aiming to characterize structural properties, explore antiproliferative activity and generate mechanistic hypotheses through experimental and computational approaches. **Methods**: Complexes were characterized by EPR, FTIR-ATR, and ESI-MS, with preliminary SC-XRD data for PH-Cu. Antiproliferative activity was evaluated against six human cancer cell lines using the MTT assay (24 h). Subcellular effects were assessed by fluorescence microscopy and RT-qPCR. Computational studies included DFT geometry optimization, target prediction, molecular docking, and ADMET profiling. **Results**: Based on spectroscopic and spectrometric data and comparison with analogous Cu(II) complexes, a distorted square-pyramidal coordination geometry was proposed; this assignment was not confirmed by SC-XRD. Both complexes exhibited potent antiproliferative activity, with PH-Cu showing the highest potency in HeLa cells (IC_50_ = 4.22 µM). Under the same conditions, cisplatin showed substantially lower activity (HepG2: 191.1 µM; Caco-2: 129.6 µM; NCI-H69: >333.3 µM; HeLa: 21.9 µM). Fluorescence microscopy at 18 h revealed pyknosis, karyorrhexis, and microtubule disorganization, consistent with regulated cell death. RT-qPCR of PH-Cu indicated intrinsic apoptotic pathway engagement (BAX +3.20-fold; BCL2 to 0.39-fold of control). DFT-optimized bond lengths were consistent with crystallographic data for analogous complexes. Molecular docking suggested PRKCG, RELA (p65), Caspase-3, and α/β-tubulin as interaction candidates, while ADMET profiling predicted favorable intestinal absorption (>92.8%) and low BBB permeability. **Conclusions**: These results suggest that the [Cu(phen)] unit constitutes the primary pharmacophore, with the dicarboxylate co-ligand as a modulator of the antiproliferative profile, suggesting promising anticancer pharmacological potential.

## 1. Introduction

Cancer remains a global health challenge characterized by uncontrolled cellular proliferation and metastatic cascades involving extracellular matrix remodeling, angiogenesis, and immune evasion [1,2]. While platinum-based drugs like cisplatin are clinical cornerstones, their application is limited by severe systemic toxicity and acquired resistance, driving the development of alternatives based on endogenous transition metals like copper [3,4]. Copper’s redox versatility (Cu^2+^/Cu^+^) and its essential role as a catalytic cofactor in energy production and ROS detoxification provide a unique opportunity for targeted therapy [5]. Among the most potent copper-based architectures are those featuring 1,10-phenanthroline (phen), a rigid hydrophobic chelator that promotes DNA intercalation and oxidative cleavage [6] whose biological efficacy exemplified by the Casiopeínas family is driven by ROS generation and topoisomerase inhibition [7,8]. In this study, we evaluate two structurally optimized complexes: PH-Cu (malonate-based) and PC-Cu (featuring 1,1-cyclobutanedicarboxylate), where the cyclobutane motif draws a strategic parallel with second-generation clinical agents like carboplatin, aiming to enhance thermodynamic stability and cellular uptake [9,10].

Despite their promising profiles, current copper(II) coordination complexes face limitations hindering clinical translation, including poor aqueous solubility, limited stability in physiological media [11], and insufficient selectivity toward cancer cells over healthy tissue [12]. The design of mixed-ligand architectures incorporating 1,10-phenanthroline alongside carboxylate co-ligands represents a strategy to modulate these properties while preserving potent antiproliferative activity.

To overcome the traditional limitations of in vitro screening, where 2D models may fail to fully replicate the complexity of the tumor microenvironment [13,14], we implement a multi-stage in silico workflow. By utilizing chemoinformatic platforms built upon machine learning (ML) and Graph Convolutional Neural Network (GCNN) architectures, it is possible to predict ADMET profiles and explore target specificity against >600 human proteins [15,16]; however, as these models were trained primarily on organic small molecules, their application to paramagnetic Cu(II) coordination complexes is exploratory and should be regarded as hypothesis-generating rather than predictive, pending experimental validation.

Herein, the objective of this work is to conduct a comprehensive investigation of PH-Cu and PC-Cu, bridging solid-state characterization and in vitro cytotoxicity with chemoinformatic target identification and gene expression profiling (RT-qPCR). By coupling DFT-optimized molecular docking with morphological assessment, this research aims to characterize the morphological and transcriptional features associated with the induced cell-death response. Furthermore, this study seeks to provide preliminary mechanistic insights how these phenanthroline-based copper(II) architectures induce a regulated cell death response, providing mechanistic insights into the structural dismantling and regulated cell death triggered in HeLa cells.

## 2. Materials and Methods

### 2.1. Chemicals

All chemicals and reagents used in this study were of analytical or spectroscopic grade purity unless otherwise specified. Copper(II) chloride dihydrate (CuCl_2_·2H_2_O, ≥99%, Sigma-Aldrich, St. Louis, MO, USA), 1,10-phenanthroline (phen, ≥99%, Sigma-Aldrich, St. Louis, MO, USA), malonic acid (≥99%, Sigma-Aldrich, St. Louis, MO, USA), cyclobutane-1,1-dicarboxylic acid (≥98%, Sigma-Aldrich, St. Louis, MO, USA), and potassium hydroxide (KOH, reagent grade, Sigma-Aldrich, St. Louis, MO, USA) were purchased from commercial suppliers and used without further purification. Double-distilled water and ethanol (absolute, ≥99.5%, Sigma-Aldrich, St. Louis, MO, USA) were employed as solvents throughout the synthesis.

For biological assays, cell lines were obtained from ATCC (American Type Culture Collection, Manassas, VA, USA): HeLa (CRM-CCL-2), HCC1954 (CRL-2338), HepG2 (HB-8065), NCI-H69 (HTB-119), Caco-2 (HTB-37), and Jurkat (TIB-152). Cells were cultured in RPMI-1640 medium supplemented with 10% fetal bovine serum (FBS) and 50 µg/mL gentamicin (both from Invitrogen, Carlsbad, CA, USA). Cisplatin (Sigma-Aldrich, St. Louis, MO, USA) was used as a positive control for cytotoxic and gene expression studies.

For molecular and cellular analyses, the following kits and reagents were employed: PureLink™ RNA Mini Kit (Invitrogen, Cat. No. 12183020, Carlsbad, CA, USA) for RNA isolation, Qubit™ RNA IQ Assay Kit (Invitrogen, Cat. No. Q33221, Carlsbad, CA, USA) for quality and integrity assessment of the RNA, and SuperScript™ III One-Step RT-PCR System with Platinum™ Taq DNA Polymerase (Invitrogen, Cat. No. 12574026, Carlsbad, CA, USA) for RT-qPCR analysis. PrimeTime™ probes (Integrated DNA Technologies, Coralville, IA, USA) labeled with FAM reporter and double-quenched with ZEN and IABk were used for gene expression quantification.

For morphological analysis, Hoechst 33342 (Invitrogen, Carlsbad, CA, USA) staining was employed for nuclear integrity assessment, monoclonal anti-α-tubulin primary antibody was used for cytoskeletal analysis, and Alexa Fluor™ 488 secondary antibody (Invitrogen, Carlsbad, CA, USA) was used for visualization. Formaldehyde (4%) was used for cell fixation. For computational studies, all molecular structures and calculations were performed using commercially available software as specified in the respective methodology sections. No additional chemical reagents were required for computational analyses.

### 2.2. Experimental Procedures and Instrumental Methods

#### 2.2.1. Synthesis of the New Copper(II) Complexes

Copper(II) coordination complexes were synthesized via a sequential two-step coordination strategy in aqueous media. First Step—Intermediate Formation: Copper(II) chloride dihydrate (0.3361 g, 2.5 mmol) was dissolved in 20 mL of double-distilled water in a round-bottom flask under constant magnetic stirring and heated to reflux at approximately 94 °C. While maintaining reflux conditions, 1,10-phenanthroline (2.5 mL of a 1.0 M solution in ethanol, 2.5 mmol, 1.0 equiv.) was added dropwise over several seconds with continuous stirring. The solution was maintained under reflux at 94 °C for an additional 10 min to ensure complete formation of the [CuCl_2_(phen)] intermediate complex, which was evidenced by a distinct color change of the solution. Second Step—Complex Formation: To prepare the dicarboxylate ligand solutions, malonic acid (for PH-Cu) or cyclobutane-1,1-dicarboxylic acid (for PC-Cu) (2.5 mmol each) were dissolved in 5 mL of double-distilled water. The dicarboxylic acid solutions were neutralized with dropwise addition of a previously prepared potassium hydroxide solution (2.5 mmol KOH in water) until neutral pH was achieved, confirmed by visual observation. The resulting potassium dicarboxylate solution was then added in its entirety to the hot [CuCl_2_(phen)] intermediate solution while maintaining reflux conditions at 94 °C. After addition, the reaction mixture was maintained under reflux for 1 h to ensure complete ligand displacement and complex formation, evidenced by a further color change.

Isolation and Drying: The reaction mixtures were allowed to cool to room temperature naturally over 24 h in a vacuum chamber to monitor crystal formation. Solid products were separated from the mother liquor by manual decantation and careful transfer of the precipitate. The solids were washed three times with small portions of cold double-distilled water with manual stirring until the washings were colorless, ensuring complete removal of water-soluble byproducts and inorganic salts. After the final washing, the solid products were dried at room temperature in a vacuum desiccator for 48 h. Both complexes were obtained as stable, microcrystalline powders exhibiting coloration with blue tones. Isolated yields of the washed microcrystalline products: PH-Cu, 95%; PC-Cu, 97% (calculated based on CuCl_2_ as the limiting reagent). The products showed no visible signs of decomposition upon storage at room temperature under atmospheric conditions during the period of study.

#### 2.2.2. FTIR-ATR Measurements

FTIR-ATR spectra were recorded in the range of 4000–450 cm^−1^ at a resolution of 2 cm^−1^ using an Agilent Technologies (Santa Clara, CA, USA) instrument (32 sample scans, Happ-Genzel apodization). Spectral assignments were based on literature values for analogous Cu(II)–phenanthroline mixed-ligand complexes. Characteristic wavenumber regions monitored for phenanthroline coordination included: aromatic C–H stretching (3000–3100 cm^−1^), C=C aromatic stretching (1300–1400 cm^−1^), C–N aromatic stretching (1050–1150 cm^−1^), and out-of-plane C–H bending modes (700–900 cm^−1^). For carboxylate ligands, asymmetric ν_as(COO^−^) stretching (1600–1700 cm^−1^) and symmetric ν_s(COO^−^) stretching (1440–1360 cm^−1^) were monitored. Free ligands were measured as reference standards. FTIR-ATR spectroscopy was employed to investigate characteristic vibrational modes and assess shifts in bands relative to free ligands. This technique supports ligand coordination through changes in characteristic vibrations but does not directly provide information about coordination geometry.

#### 2.2.3. ESI-MS Analysis

Electrospray ionization mass spectrometry was employed to determine the molecular composition and characterize fragmentation patterns of the synthesized complexes. ESI-MS analysis was performed at Instituto Transdisciplinar de Investigación y Servicios (ITRANS) using a UPLC-MS system equipped with an electrospray ionization (ESI) source and a quadrupole time-of-flight (Q-TOF) mass analyzer providing a mass resolving power of 50,000 FWHM (Full Width at Half Maximum(Waters, Milford, MA, USA), enabling high-resolution mass measurement and accurate mass determination. Samples were dissolved in DMSO and analyzed via direct infusion. Mass spectra were acquired in both positive and negative electrospray ionization modes (ESI+ and ESI−) using the following parameters: capillary voltage ±3.5 kV, cone voltage 25–30 V, source temperature 120–150 °C, *m*/*z* acquisition range 150–600, scan mode with acquisition rate 0.2–1 spectrum/s, and individual spectra averaged from ≥40 accumulated scans. MS/MS fragmentation patterns were obtained using collision-induced dissociation (CID). The characteristic copper isotopic pattern (^63^Cu/^65^Cu, intensity ratio ~1.3:1) was used to identify copper-containing ions. Fragmentation patterns were interpreted according to established protocols for Cu(II)–phenanthroline mixed-ligand complexes. ESI-MS confirms molecular mass and composition of the complexes. However, this technique does not directly provide information about coordination geometry. ESI-MS is a spectrometric technique (not spectroscopic), and ionization processes may cause partial ligand exchange or complex fragmentation in the gas phase.

#### 2.2.4. EPR Measurements

Electron Paramagnetic Resonance (EPR) spectra were recorded to investigate the electronic structure and coordination environment of Cu(II). X-band EPR measurements were performed at Instituto Transdisciplinar de Investigación y Servicios (ITRANS) using a JEOL JES-FA200 spectrometer (JEOL’s, Akishima, Tokyo, Japan) operating at a microwave frequency of 9.5–9.65 GHz (X-band). The following acquisition parameters were employed: microwave power 2–10 mW, modulation frequency 100 kHz, field modulation amplitude optimized for each sample (1.0 mT for CuCl_2_, 0.05 mT for PH-Cu, 0.35 mT for PC-Cu, and 0.01 mT for [Cu(phen)Cl_2_]), magnetic field range 0–500 mT, time constant 5–10 ms, receiver gain optimized for signal quality, and measurement temperature 298 K (ambient). Spectra were recorded for the CuCl_2_ precursor, the intermediate [Cu(phen)Cl_2_] complex, and the final complexes PH-Cu and PC-Cu (2–4 accumulated field sweeps per sample). Extracted g-values were compared to literature values for analogous Cu(II)–phenanthroline systems. EPR spectroscopy supports the characterization of the Cu(II) electronic environment but does not unambiguously determine coordination geometry without complementary evidence from other techniques.

### 2.3. Biological Evaluation

#### 2.3.1. In Vitro Cytotoxicity and Apoptotic Morphology Analysis

The cytotoxic activity of PH-Cu and PC-Cu was evaluated against a panel of six human cancer cell lines (HeLa, HCC1954, Jurkat, HepG2, Caco-2, and NCI-H69) (American Type Culture Collection, Manassas, VA, USA) using the MTT assay. Cells were seeded in 96-well plates (2 × 10^4^ cells/well) in supplemented RPMI-1640 medium and treated with serial concentrations of the complexes for 24 h. Stocks solutions were prepared in mg/L, and the corresponding working concentrations were converted to µM using each compound’s molecular weight. The final concentration of DMSO in the culture media did not exceed 0.5% (*v*/*v*), and this concentration was verified to have no significant effect on cell viability. Cell viability at each concentration was determined by comparing absorbance values to untreated controls, which were considered to have 100% viability. Cisplatin was used as the reference metallodrug under the same 24 h exposure protocol as the test compounds.

The initial MTT screening was performed as an exploratory dose-finding assay to estimate the working concentrations required for the subsequent high-resolution molecular and morphological evaluations. Cell viability data from four technical replicates per concentration within a single representative screening plate were fitted to a constrained sigmoidal four-parameter logistic (4PL) non-linear regression model (fixed limits: 0% and 100% viability) using the drc package in R. The estimated half-maximal inhibitory concentrations (IC_50_) and their 95% confidence intervals (95% CI) derived from this 4PL model were utilized to guide the dosing of downstream assays, and should be regarded as working concentrations rather than definitive pharmacological endpoints. Subsequently, subcellular damage was evaluated in HeLa cells after 18 h of exposure at the working concentration derived from the initial MTT screening (PH-Cu, 1.23 mg/L, 3.38 µM). Nuclear integrity was assessed using Hoechst 33342 (Invitrogen, Carlsbad, CA, USA) staining to detect apoptotic hallmarks, including chromatin condensation and nuclear fragmentation. Simultaneously, cytoskeletal organization was analyzed through α-tubulin immunocytochemistry; cells were fixed with 4% formaldehyde and incubated with a monoclonal anti-α-tubulin primary antibody, followed by an Alexa Fluor™ 488 secondary antibody. Morphological alterations were visualized using fluorescence microscopy with Hoechst 33342 (Invitrogen, Carlsbad, CA, USA) and Alexa Fluor™ 488 (Invitrogen, Carlsbad, CA, USA) signals using via DAPI and FITC channels, respectively. This imaging was conducted as a qualitative phenotypic validation; multiple random fields of view were visually screened to ensure uniform population responses prior to capturing representative single-field images. Formal manual or automated quantification of the morphological hallmarks was not performed in this exploratory phase.

#### 2.3.2. Gene Expression Analysis via RT-qPCR

HeLa cervical adenocarcinoma cells (ATCC, Manassas, VA, USA) were cultured in RPMI-1640 medium supplemented with 10% fetal bovine serum and 50 µg/mL gentamicin (Invitrogen, Carlsbad, CA, USA). Cells were seeded at a density of 2 × 10^4^ cells per well in 96-well culture plates and incubated at 37 °C in a humidified atmosphere with 5% CO_2_ for 24 h to ensure adherence. Cultures were subsequently treated for 12 h with PH-Cu and PC-Cu at the working concentrations derived from the initial MTT screening (PH-Cu, 1.23 mg/L, 3.38 µM; PC-Cu, 2.07 mg/L, 5.36 µM), alongside cisplatin (6.30 mg/L, 20.99 µM) as a reference. These working concentrations correspond to the exploratory dose-finding estimates used to guide the downstream assays and are not identical to the refined IC_50_ values reported in results (Section 3.5), which were subsequently recalculated using the constrained 4PL model. Untreated cells in pure culture media were maintained in parallel as the calibrator condition. The absence of a matched DMSO vehicle control (≤0.5% *v*/*v*) for normalization is acknowledged as a limitation of this exploratory transcriptomic design. Following treatment, cells were harvested using a cell lifter, and total RNA was isolated using the PureLink™ RNA Mini Kit (Invitrogen, Cat. No. 12183020, Carlsbad, CA, USA) according to the manufacturer’s instructions. RNA concentration and integrity were verified fluorometrically using the Qubit™ RNA IQ Assay Kit on a Qubit 4 Fluorometer (Invitrogen, Cat. No. Q33221, Carlsbad, CA, USA). An electrophoretic integrity measurement (Bioanalyzer/TapeStation RIN) was not performed.

Relative gene expression was quantified via one-step RT-qPCR using the SuperScript™ III One-Step RT-PCR System with Platinum™ Taq DNA Polymerase (Invitrogen, Cat. No. 12574, Carlsbad, CA, USA). Fluorescence signaling was achieved using PrimeTime™ (IDT) probes (FAM reporter, double-quenched with ZEN and IABk). Target genes included BAX, BCL2, DAXX, NFKB1, EIF4E, and SOD1, with GAPDH as the endogenous reference. Reactions (25 µL) were performed on a CFX96 Touch Real-Time PCR Detection System (Bio-Rad Laboratories, Hercules, CA, USA) following optimized thermal profiles. All samples were run in technical triplicate, including no-template (NTC) and no-reverse-transcription (–RT) controls, which were confirmed clean with no detectable amplification. Amplification efficiencies were previously verified via standard curves to fall within the acceptable 90–110% range across a dynamic range of 4 logs, validating the use of the 2^−ΔΔCt^ method for relative quantification. Primer and probe sequences for all targets are provided in Supplementary Table S1.

Statistical significance was assessed by one-way ANOVA followed by Dunnett’s post-hoc test for comparisons against the untreated control. Statistical testing was performed on log_2_-transformed fold change values to satisfy normality assumptions. Data are presented as mean ± SEM from independent biological replicates (*n* = 3 per group for all statistically evaluated conditions: untreated control, PH-Cu and cisplatin). The *n* = 2 sample size allocation was applied strictly to the exploratory PC-Cu treatment, which exhibited a large reference gene (GAPDH) amplification shift relative to the control. This instability precluded reliable 2^−ΔΔCt^ normalization; therefore, PC-Cu was completely excluded from quantitative fold-change analysis and ANOVA modeling, and is reported only descriptively as raw Cq values (Figure S1). Differences were considered significant at *p* < 0.05. All analyses were performed in R (v4.4.1).

### 2.4. Computational Methods

#### 2.4.1. DFT Geometry Optimization

The electronic structure and geometries of the Cu(II) complexes were investigated via Density Functional Theory (DFT) using ORCA 5.0 [17] and Avogadro 1.2.0 to design the molecular structure [18]. Initial structures were pre-optimized with the Universal Force Field (UFF) and subsequently refined in the gas phase using the B3LYP hybrid functional. A mixed basis set strategy was applied: def2-TZVP for the Cu(II) center and def2-SVP for light atoms (C, H, N, O). To account for dispersion effects, the Grimme’s D3 correction with Becke-Johnson damping (D3BJ) was incorporated. All systems were treated with a doublet spin multiplicity (M = 2). Harmonic vibrational frequency calculations confirmed the absence of imaginary frequencies, ensuring true local minima.

#### 2.4.2. Machine Learning-Based Target Prediction

A multi-stage in silico pipeline was implemented using SMILES notation and molecular graphs representations generated with RDKit and OpenBabel. Structural identifiers and molecular representation were checked using CACTUS [19], while ADMET-related properties were predicted using ADMETlab 3.0 [20] and PkCSM [21]. Potential therapeutic targets were identified through a dual-server consensus (SuperPred [22]/TargetNet [23]) utilizing ChEMBL v29 data and QSAR models against >600 human proteins. Biological roles and oncogenic associations (e.g., apoptosis evasion, angiogenesis) were mapped via GeneCards [24] and UniProt [25]. The workflow integrated chemoinformatic descriptors, molecular graph-based structural signatures, and machine learning approaches, including Graph Convolutional Neural Network (GCNN)-based models where available.

It should be noted that SuperPred, TargetNet, and the underlying QSAR/ML models were trained primarily on organic small molecules; their applicability to a paramagnetic d^9^ Cu(II) complex with a labile coordination sphere remains unvalidated. Target predictions should therefore be regarded as hypothesis-generating rather than definitive, pending experimental confirmation.

#### 2.4.3. Molecular Docking Simulations

Interaction mechanisms were explored against key apoptotic and structural targets from the RCSB Protein Data Bank: PRKCG (PDB ID: 2I0E) [26], BAX (PDB ID: 2YXJ) [27], BCL2 (PDB ID: 2W3L) [28], MDM2 (PDB ID: 1T4E) [29], Caspase-3 and 9 (PDB ID: 3KJF- PDB ID: 1NW9) [30,31], α/β-tubulin(PDB ID: 1SA0) [32], NF-κB (p50 subunit) and RELA (p65 subunit) (PDB ID: 1VKX) [33]. Receptors were prepared in BIOVIA Discovery Studio v2025 and AutoDock Tools v4 [34,35] by removing co-crystallized ligands, crystallographic water molecules and non-essential heteroatoms, followed by the addition of polar hydrogen atoms and applying Kollman partial charges. Simulations were executed in AutoDock Vina (exhaustiveness: 8; spacing: 0.375 Å) using DFT-optimized ligand geometries. Search grids were centered on catalytic or interaction sites, and binding affinities (ΔG, kcal/mol) were recorded.

It should be noted that the AutoDock Vina scoring function is parameterized for organic ligands and does not explicitly treat copper coordination chemistry; accordingly, the reported ΔG values should be interpreted as relative, hypothesis-generating rankings rather than quantitative binding affinities. Furthermore, these simulations assume that the coordination sphere of the Cu(II) complexes remains rigid and intact upon target binding; in practice, Cu(II) complexes may undergo partial ligand exchange with coordinating amino-acid residues, so the predicted binding poses should be regarded as approximations of the bound species, pending experimental validation.

## 3. Results and Discussion

### 3.1. Synthesis and General Characteristics of Copper(II) Complexes

The synthesis of PH-Cu and PC-Cu was achieved with near-quantitative molar yields of 95% and 97%, respectively. Both complexes were obtained as microcrystalline powders exhibiting blue-toned coloration, with no visible signs of decomposition upon storage at room temperature.

**Figure 1:**
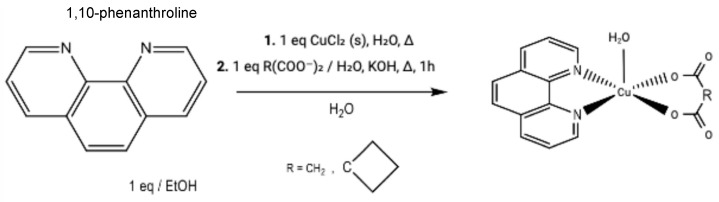
Synthetic route for the preparation of PH-Cu and PC-Cu coordination complexes via sequential stoichiometric coordination in aqueous media.

As illustrated in the scheme (Figure 1), both complexes were obtained via a sequential two-step coordination strategy in aqueous media. In the first step, CuCl_2_ was dissolved in double-distilled water and heated to boiling, after which 1,10-phenanthroline (1 eq, 1 M in ethanol) was added and stirred for 10 min to promote the formation of the [Cu(phen)Cl_2_] intermediate, where the bidentate N,N-donor ligand occupies two coordination sites of the Cu(II) center. In the second step, the corresponding dicarboxylate ligand (1 eq, 1 M aqueous solution, neutralized with KOH) was incorporated under reflux conditions for 1 h, likely displacing the two chloride ions and consistent with completion of the proposed square-pyramidal coordination sphere through bidentate O,O-coordination. For PH-Cu, the malonato ligand (R = CH_2_) coordinates through its two carboxylate oxygen atoms via a flexible methylene bridge, yielding a trans configuration around the Cu(II) center. For PC-Cu, the cyclobutane-1,1-dicarboxylate ligand (R = cyclobutane) imposes a more rigid geometric constraint due to the cyclic carbon framework, resulting in a cis configuration of the carboxylate oxygen atoms. Cooling to room temperature induced precipitation of the solid products, which were recovered by manual decantation, washed with cold double-distilled water, and dried to constant weight.

As a preliminary structural approach, the molecular structure of PH-Cu was investigated by single-crystal X-ray diffraction analysis, revealing a triclinic crystal system with two independent coordination entities per unit cell and three non-coordinated crystallization water molecules. In both metal units, the copper center presents a coordination number of five, coordinated to one 1,10-phenanthroline molecule, one bidentate malonate anion, and one axial water molecule, generating a CuN_2_O_3_ chromophore with a distorted square-pyramidal geometry, consistent with the crystallographic description reported by Diallo et al. [36] for the analogous aquamalonato(1,10-phenanthroline)copper(II) system. Selected crystal parameters and bond distances are provided in Supplementary Tables S2 and S3; the corresponding diffraction pattern is shown in Supplementary Figure S1. It should be noted that this analysis represents a preliminary crystallographic characterization; a more comprehensive structural determination, including full refinement parameters and high-resolution data, as well as an analogous SC-XRD analysis for PC-Cu, remain important priorities for future work to fully establish the solid-state structures of both complexes.

### 3.2. FTIR-ATR Spectroscopic Characterization

The successful formation of both complexes was supported by FTIR-ATR spectroscopy. The spectra of both PH-Cu and PC-Cu indicated the presence of the coordinated ligands through their characteristic vibrational bands (Table 1). In both PH-Cu and PC-Cu, the aromatic C–H stretching (signal A, 3000–3100 cm^−1^), C=C aromatic stretching (signal C, 1300–1400 cm^−1^), C–N aromatic stretching (signal D, 1050–1150 cm^−1^), and the out-of-plane C–H bending modes of the central ring (signal E, 800–900 cm^−1^) and the heterocyclic ring (signal G, 700–750 cm^−1^) are consistent with the presence of coordinated 1,10-phenanthroline. In both complexes, the carboxylate asymmetric stretching ν_as_(COO^−^) (signal B, 1600–1700 cm^−1^) appears shifted to lower wavenumbers relative to the corresponding free dicarboxylic acid ν(C=O) (~1710 cm^−1^), supporting deprotonation and bidentate O,O-coordination of the dicarboxylate ligand to the Cu^2+^ center. Additionally, both complexes display a distinctive band at 750–800 cm^−1^ (signal F), assigned to skeletal ring vibrations of the coordinated ligands.

These spectral shifts are consistent with those reported by Abebe and Hailemariam [37] for Cu(II)–phenanthroline coordination complexes. The aromatic C–H stretching (signal A, 3000–3100 cm^−1^) is consistent with C–H stretching vibrations of the phenanthroline ligand reported in the 3020–3090 cm^−1^ region. The carboxylate C=O stretching (signal B, 1540–1650 cm^−1^) indicates coordination of the dicarboxylate ligand to the Cu(II) center. The C=C aromatic stretching (signal C, 1350–1500 cm^−1^) is consistent with C–C stretching modes reported near 1327–1431 cm^−1^ for the phenanthroline ring system.

It should be noted that the localized νC=N and νC=C stretching modes of coordinated 1,10-phenanthroline have been reported at higher wavenumbers (1626 and 1587 cm^−1^, respectively) by Wang et al. [41]; these modes likely fall within the envelope of signal B (1600–1700 cm^−1^) and were not resolved as distinct bands in our spectra. Accordingly, signal C is more accurately attributed to aromatic ring skeletal stretching modes of the phenanthroline framework rather than a localized C=C stretch.

The C–N aromatic stretching (signal D, 1050–1150 cm^−1^) further supports coordination of the nitrogen atoms to the metal center. The bands observed in the 700–900 cm^−1^ region (signals E and G) fall within the range reported for out-of-plane vibrational modes of the phenanthroline ring system in analogous Cu(II)–phenanthroline complexes.

**Figure 2:**
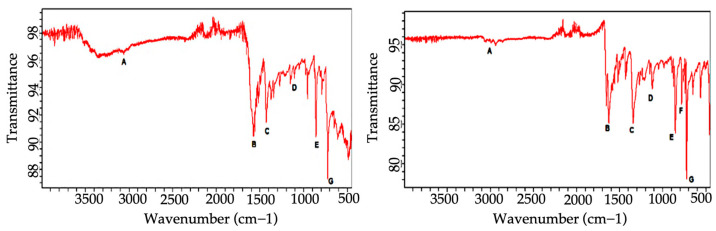
FTIR-ATR spectra of the synthesized copper(II) complexes: PH-Cu (**left**) and PC-Cu (**right**). Characteristic vibrational bands are labeled: (A) aromatic C–H stretching (3000–3100 cm^−1^), (B) carboxylate asymmetric stretching ν_as_(COO^−^) (1600–1700 cm^−1^), (C) aromatic C=C stretching (1350–1500 cm^−1^), (D) aromatic C–N stretching (1050–1150 cm^−1^), (E) out-of-plane C–H bending of the phenanthroline ring (800–900 cm^−1^), (F) skeletal ring vibration of coordinated ligands (750–800 cm^−1^, present in both PH-Cu and PC-Cu), and (G) out-of-plane C–H bending of the phenanthroline heterocyclic ring (700–750 cm^−1^).

Supporting these observations, the FTIR-ATR data obtained for PH-Cu and PC-Cu (Figure 2) are consistent with those reported by Chavelas-Hernández et al. [42] for analogous Cu(II) mixed complexes containing 1,10-phenanthroline as the primary ligand. Specifically, the carboxylate stretching band (signal B, 1600–1700 cm^−1^) appears shifted to lower wavenumbers relative to the free dicarboxylic acid ν(C=O) (~1710 cm^−1^), in agreement with the ν(C=O) values of 1567–1568 cm^−1^ reported for coordinated carboxylate groups in Cu(II)–phenanthroline complexes, supporting deprotonation and O,O-bidentate coordination of the dicarboxylate ligand to the Cu^2+^ center. Additionally, the out-of-plane C–H bending band of the heterocyclic ring (signal G, 700–750 cm^−1^) is consistent with the C–H bending bands at 721–723 cm^−1^ assigned to the 1,10-phenanthroline ligand in related Cu(II) complexes, supporting the presence of the coordinated phenanthroline ligand in both synthesized compounds. On the other hand, the shift of the carboxylate asymmetric stretching band (signal B) to lower wavenumbers relative to the free dicarboxylic acid ν(C=O) (~1710 cm^−1^) is consistent with the coordination of the carboxylate group to the Cu^2+^ center. According to Justi et al. [38], the asymmetric COO^−^ stretching vibrations in metal–carboxylate complexes appear in the range of 1620–1540 cm^−1^, with the shift of this band relative to the free acid being indicative of metal–carboxylate coordination. The separation between the asymmetric ν_as_(COO^−^) and symmetric ν_s_(COO^−^) stretching bands, known as Δν(COO^−^), has been widely used to assess the coordination mode of carboxylate groups following the criteria established by Deacon and Phillips [43]. The observed shift in signal B for both PH-Cu and PC-Cu is consistent with the deprotonation of the dicarboxylic acid and its coordination to Cu^2+^ through the carboxylate oxygen atoms.

### 3.3. ESI-MS-Based Molecular Analysis

**Figure 3:**
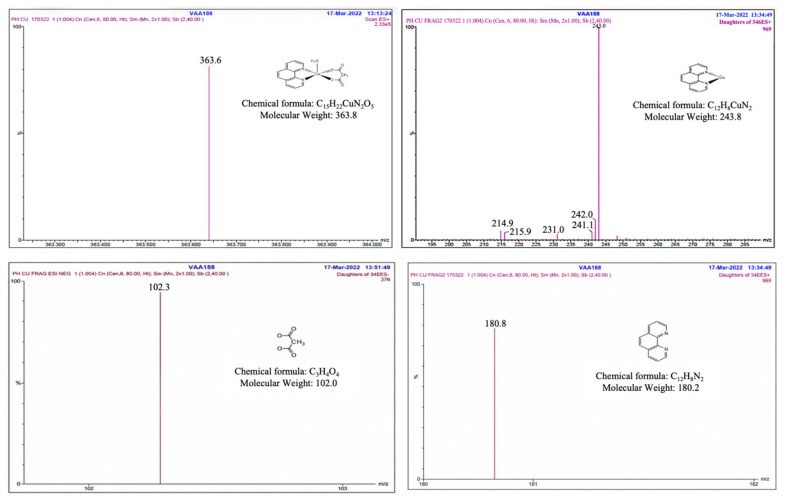
Electrospray ionization mass spectra (ESI-MS) of PH-Cu.

The ESI-MS spectrum of PH-Cu (Figure 3) in positive ion mode showed the molecular ion at *m*/*z* = 345.8, corresponding to the anhydrous complex [Cu(phen)(mal)] (C_15_H_10_CuN_2_O_4_, molecular weight 345.8 g/mol), and a second ion at *m*/*z* = 363.6, assigned to the monohydrated species [Cu(phen)(mal)(H_2_O)] (C_15_H_12_CuN_2_O_5_, molecular weight 363.8 g/mol). MS/MS fragmentation in positive mode yielded the base peak at *m*/*z* = 243.0, assigned to [Cu(phen)]^+^ (C_12_H_8_CuN_2_, molecular weight 243.8 g/mol), displaying the characteristic copper isotopic pattern (*m*/*z* = 243 and 245, corresponding to ^63^Cu and ^65^Cu, respectively), supporting coordination of the metal center to 1,10-phenanthroline. A fragment at *m*/*z* = 180.2 corresponding to free 1,10-phenanthroline (C_12_H_8_N_2_, molecular weight 180.2 g/mol) was also observed. In negative ion mode, a peak at *m*/*z* = 102.3 was identified and assigned to the free malonate dianion [CH_2_(COO^−^)_2_] (C_3_H_2_O_4_, molecular weight 102.0 g/mol), supporting incorporation of the dicarboxylate ligand into the complex. Collectively, these data support the successful synthesis of [Cu(phen)(mal)(H_2_O)].

**Figure 4:**
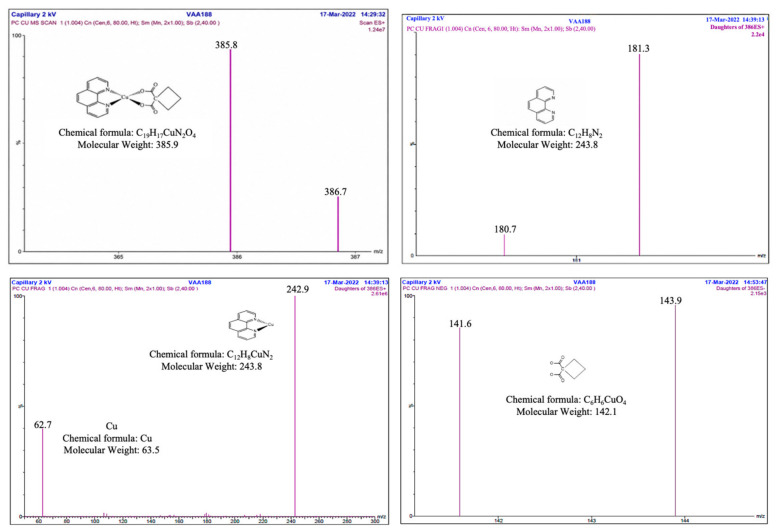
Electrospray ionization mass spectra (ESI-MS) of PC-Cu.

For PC-Cu (Figure 4), the ESI-MS spectrum in positive ion mode displayed the molecular ion at *m*/*z* = 385.8, consistent with the anhydrous complex [Cu(1,10-phenanthroline)(cyclobutane-1,1-dicarboxylate)] (C_18_H_14_CuN_2_O_4_, molecular weight 385.9 g/mol), along with its ^65^Cu isotopologue at *m*/*z* = 386.7. MS/MS fragmentation in positive mode yielded the base peak at *m*/*z* = 242.9, assigned to [Cu(1,10-phenanthroline)]^+^ (C_12_H_8_CuN_2_, molecular weight 243.8 g/mol), and a minor fragment at *m*/*z* = 62.7 corresponding to free Cu^+^ (molecular weight 63.5 g/mol), further supporting the presence of the copper center. A fragment at *m*/*z* = 181.3 corresponding to free 1,10-phenanthroline (C_12_H_8_N_2_, molecular weight 180.2 g/mol) was also observed. In negative ion mode, a peak at *m*/*z* = 143.9 was identified and assigned to the cyclobutane-1,1-dicarboxylate dianion (C_6_H_6_O_4_, molecular weight 142.1 g/mol), supporting the incorporation of this ligand into the complex. Collectively, these data support the successful synthesis of [Cu(1,10-phenanthroline)(cyclobutane-1,1-dicarboxylate)].

MS/MS fragmentation in positive ion mode for both PH-Cu and PC-Cu showed the most intense peaks at *m*/*z* 243.0 and 242.9, respectively. These peaks were assigned to the [Cu(phen)]^+^ fragment ion (C_12_H_8_CuN_2_^+^), which contains copper and one 1,10-phenanthroline ligand. The peak at *m*/*z* ≈ 243 corresponds to the fragment containing the ^63^Cu isotope. The characteristic copper isotopic pattern should display another peak at approximately *m*/*z* 245, corresponding to the ^65^Cu isotope, with an intensity ratio of roughly 2.2:1. The weak peaks observed at *m*/*z* 241 and 242, each with relative intensity below 10%, likely represent minor fragment peaks or background signals rather than the main copper isotopic pattern. The identification of copper through its isotopic signature in ESI-MS has been established as a reliable approach for confirming the presence of the metal center in coordination complexes, as demonstrated by Tsednee et al. [44]. A fragment corresponding to free 1,10-phenanthroline was also observed in both cases, at *m*/*z* = 180.2 for PH-Cu and *m*/*z* = 181.3 for PC-Cu. The [Cu(phen)]^+^ fragment at *m*/*z* = 243 and the free phenanthroline fragment [phen+H]^+^ at *m*/*z* = 181 are consistent with fragmentation products previously reported by Pivetta et al. [45] for Cu(II)–phenanthroline complexes, where both species were identified as characteristic ions arising from the fragmentation of the Cu–phen bond during MS/MS experiments. Furthermore, the presence of [Cu(phen)]^+^ as a common MS/MS fragment in both complexes is consistent with results reported by Masuri et al. [46] for analogous Cu(II)–phenanthroline mixed complexes, supporting the role of 1,10-phenanthroline as the primary coordinating ligand in both synthesized compounds.

NMR spectroscopy was not applicable owing to the paramagnetic d^9^ Cu(II) center, which precludes acquisition of well-resolved spectra; structural assignment therefore relied on FTIR-ATR, ESI-MS, EPR, and DFT.

The solution stability of PH-Cu and PC-Cu under physiological conditions was not directly assessed in this study and represents a limitation to be addressed in future work through time-resolved UV-Vis spectroscopy or ESI-MS monitoring in biological media. Both complexes were dissolved in DMSO and diluted in cell culture medium immediately prior to use, a protocol widely employed for Cu(II) coordination compounds to minimize hydrolysis. Indirect evidence of sufficient stability is provided by the reproducible dose-dependent antiproliferative responses across all six cell lines and the distinct transcriptional and morphological effects documented at 12–18 h, which are inconsistent with rapid complex decomposition prior to cellular uptake.

### 3.4. Solid-State EPR Spectroscopic Characterization

**Figure 5:**
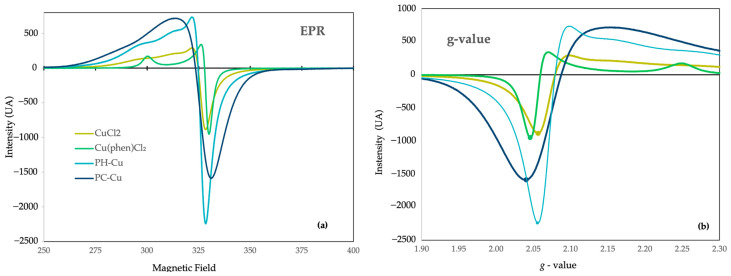
EPR derivative spectra (**a**) and g-value correlation (**b**) for the synthesized copper(II) architectures. Data are presented with a vertical offset to facilitate the comparison of the axial signals. (**a**) EPR derivative spectra recorded at room temperature for the precursor (CuCl_2_), the intermediate ([Cu(phen)Cl_2_]), and the final architectures of PH-Cu and PC-Cu. (**b**) g-value correlation for the same four samples shown in panel (**a**), highlighting the well-defined axial signals (g∥ > g⊥ > 2.0023). The spectra exhibit a smooth envelope curve lacking hyperfine splitting, a feature attributed to magnetic concentration and dipolar broadening in the solid state.

The solid samples were analyzed by solid-state EPR spectroscopy to obtain information on the electronic environment of the Cu(II) centers and to support the proposed coordination geometry. The powder EPR spectra of the complexes PH-Cu and PC-Cu (Figure 5) exhibit a well-defined axial signal with g∥ = 2.097 and g⊥= 2.080 for PH-Cu, and g∥ = 2.115 and g⊥ = 2.087 for PC-Cu. Following the established assignment criterion for Cu(II) complexes, the relationship g∥ > g⊥ > 2.0023 indicates that the unpaired electron resides predominantly in the dx^2^ − y^2^ orbital, a configuration characteristic of (square-pyramidal) coordination environments.

Notably, the spectra lack resolved hyperfine splitting, appearing as a smooth envelope curve. This feature is attributed to magnetic concentration and dipolar broadening in the solid state, as previously described by Martins et al. [47] for the [CuCl_2_(phen)] system. This interpretation was not directly verified in the present study; confirmation would require dilution of the sample in a diamagnetic Zn(II) matrix or measurement in frozen solution, both of which represent limitations of the current characterization to be addressed in future work. Consequently, A∥ values and the associated Peisach–Blumberg g∥/A∥ ratio could not be obtained, and spectral simulation (e.g., using EasySpin) was not performed in this study.

Furthermore, our experimental values for the perpendicular component (g⊥ = 2.080–2.087) are congruent with those reported by Paliwal et al. [48] for mononuclear Cu(II)-phenanthroline systems (g⊥ =2.0739). The presence of the planar 1,10-phenanthroline ligand and the axial symmetry of both complexes are structural features previously associated with DNA intercalative binding modes in related Cu(II)-phen systems, as documented by Chikira et al. [49] where auxiliary ligands modulate the stability and orientation of the complex–target interface.

While the structural features described above are consistent with DNA intercalative binding modes reported for related Cu(II)–phenanthroline complexes, the experimental evidence presented in this study (Section 3.5) suggests a predominantly protein-targeted apoptotic mechanism for PH-Cu and PC-Cu. As discussed in Section 3.5, the comparable cytotoxic potency of PH-Cu, PC-Cu, and the dicarboxylate-free intermediate Cu(phen)Cl_2_ indicates that the [Cu(phen)] unit constitutes the primary pharmacophore, with the anionic co-ligands acting mainly as haptophores that modulate physicochemical properties rather than as primary determinants of the biological mechanism. This interpretation is consistent with the selective BAX/BCL2 modulation observed in Section 3.6, which lacks the global transcriptional suppression characteristic of DNA intercalating agents, and with the molecular docking results in Section 3.10, which identified PRKCG, Caspase-3, and α/β-tubulin as primary predicted interaction sites for the Cu(phen) pharmacophore.

### 3.5. Cytotoxic Activity and Apoptotic Morphology

**Figure 6:**
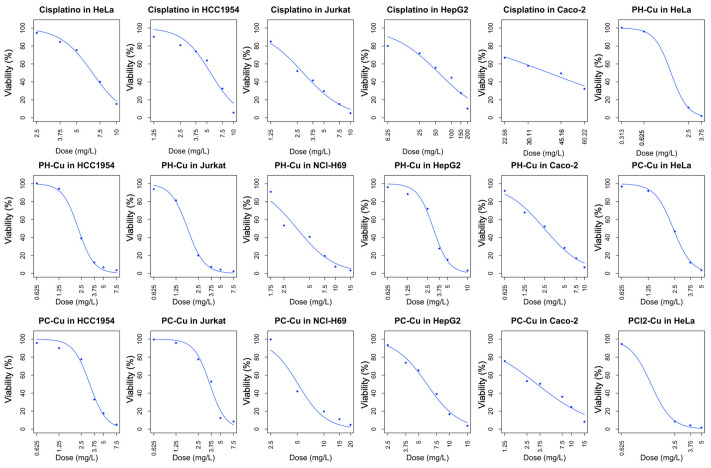
Dose–response curves of the synthesized coordination complexes and cisplatin across the evaluated human cancer cell lines. Cell viability data from technical replicates were fitted to a constrained sigmoidal four-parameter logistic (4PL) non-linear regression model (fixed limits: 0% and 100% viability). Note: The x-axis displays the working doses in mg/L, which were subsequently converted to µM using each compound’s molecular weight to estimate the working IC_50_ values reported in Supplementary Table S4.

The cytotoxic evaluation of PH-Cu and PC-Cu across six cancer cell lines identified PH-Cu as the most potent antiproliferative agent. Following the 4PL non-linear regression analysis, the estimated working IC_50_ values and 95% confidence intervals (95% CI) for all complexes were determined as summarized in Table 2. HeLa cells were the most sensitive to both complexes, yielding estimated IC_50_ values of 4.22 µM (95% CI: 3.79–4.63 µM) and 6.22 µM (95% CI: 5.78–6.63 µM) for PH-Cu and PC-Cu, respectively, highlighting the critical role of the malonate ligand framework in optimizing the antiproliferative efficacy of the copper center. The dose–response profiles for each compound across all tested cancer cell lines are illustrated in Figure 6.

Both complexes significantly outperformed cisplatin, which exhibited negligible activity (IC_50_ > 128.5 µM) against HepG2 and Caco-2 cell lines, while PH-Cu and PC-Cu maintained high potency with IC_50_ values below 15.7 µM across all tested lines. Notably, PH-Cu showed an approximately 5-fold lower IC_50_ than cisplatin in HeLa under the present 24 h protocol; this margin is cell-line- and assay-time-dependent and is largest in lines with documented intrinsic cisplatin resistance [50], rather than representing an absolute potency ranking. Moreover, PH-Cu (4.22 µM) exhibited nearly ten-fold higher potency compared to the Cu(II)–phenanthroline complex reported by Babić et al. [51] (IC_50_ = 29.8 µM at 24 h) in the same cell model, underscoring a superior optimization of the coordination environment relative to previously reported analogues.

The IC_50_ value obtained for PH-Cu (4.22 µM) in HeLa cells is consistent with the activity range reported for analogous Cu(II)–phenanthroline mixed complexes, such as the complex reported by Paliwal et al. [48] (IC_50_ = 2.63 µM in HeLa cells), further supporting the antiproliferative potential of mono-phenanthroline Cu(II) coordination compounds in this cell line. Notably, an exploratory dose-finding screening showed that the individual precursors CuCl_2_, 1,10-phenanthroline, and the dicarboxylic acids exhibited no significant cytotoxicity at the concentrations evaluated (>1109 µM), while PH-Cu, PC-Cu, and the reference compound [Cu(phen)Cl_2_] showed comparable screening activity in HeLa cells (Supplementary Table S4), consistent with findings reported by Paliwal et al. [48], who demonstrated that free ligands had negligible cytotoxic effects compared to the intact Cu(II)–phenanthroline complex at the same concentration. These results collectively suggest that the pharmacophore responsible for the antiproliferative activity resides in the [Cu(phen)] coordination unit, where 1,10-phenanthroline acts as the primary bidentate N,N-donor ligand, while the anionic co-ligands function as haptophores modulating the physicochemical properties of the complex.

Against HepG2 cells, PH-Cu (IC_50_ = 8.82 µM) and PC-Cu (IC_50_ = 15.63 µM) demonstrated superior antiproliferative activity compared to the phenanthroline derivative (compound 5) reported by Li et al. [41] (IC_50_ = 97 ± 2 µM), representing a 12-fold and 6-fold improvement in potency, respectively. Furthermore, despite their mono-phenanthroline architecture, both complexes achieved antiproliferative effects comparable to bis-phenanthroline systems reported by Shi et al. [52], who employed [Cu(phen)_2_]^2+^ cores with mitochondrial targeting vectors. This efficiency challenges the trend established by Pivetta et al. [45] and Chen et al. [53], where cytotoxicity typically scales with the number of coordinated phenanthroline units, suggesting that the incorporation of a dicarboxylate co-ligand in a single-phenanthroline scaffold may represent a viable and simplified alternative for maintaining elevated antiproliferative activity. Since the initial MTT screening was conducted using technical replicates to establish a baseline, the derived IC_50_ values are considered to be estimated working concentrations rather than absolute pharmacological endpoints. Nevertheless, these estimated values provided a reliable dose threshold that successfully triggered the cytoskeletal remodeling and transcriptional apoptotic hallmarks evaluated downstream, validating the biological relevance of the selected concentrations.

Overall, the superior antiproliferative activity of PH-Cu and PC-Cu relative to cisplatin is consistent with the general trend reported for Cu(II)–dicarboxylate–phenanthroline complexes by O’Sullivan et al. [54], where all metal–phenanthroline complexes exhibited IC_50_ values below 10 µM compared to 40.10 µM for cisplatin in cancer cell lines. Direct comparison of IC_50_ values across studies should be interpreted with caution, as cytotoxicity is strongly influenced by assay format, exposure time, and the specific cell-line batch.

The absence of significant cytotoxic activity of cisplatin in NCI-H69 cells (which exceeded the maximum evaluated concentration, resulting in an extrapolated IC_50_ > 333.3 µM) under the experimental conditions warrants specific consideration. NCI-H69 is a small cell lung cancer (SCLC) cell line that, despite belonging to a tumor type historically considered chemosensitive to platinum-based regimens, exhibits well-documented mechanisms of intrinsic and acquired cisplatin resistance. Specifically, Liang et al. [55] demonstrated that NCI-H69-derived cells can develop resistance through significant upregulation of *TRIB2*, with resistant cells exhibiting IC_50_ values up to 7-fold higher than parental cells. Furthermore, intrinsic PD-L1/PD1 signaling has been identified as a predictor of poor cisplatin efficacy in aggressive small cell lung cancer (SCLC) models [56]. Additionally, the 24 h exposure window employed in the present MTT assay may represent an insufficient timeframe to capture the full cytotoxic response of cisplatin in this cell line, as platinum–DNA adduct formation and downstream apoptotic signaling typically require longer incubation periods. The elevated cisplatin IC_50_ in HepG2 (191.1 µM) is likewise consistent with the well-documented intrinsic cisplatin resistance of this hepatocellular carcinoma cell line, mediated by high intracellular glutathione and elevated glutathione-S-transferase and efflux-transporter activity that sequester and extrude platinum species [50]. As with NCI-H69, the 24 h exposure window may further underestimate cisplatin potency, since platinum–DNA adduct formation and downstream signaling typically require longer incubation. These values should therefore be read as condition-specific reference points under a uniform 24 h protocol rather than as absolute potencies.

**Figure 7:**
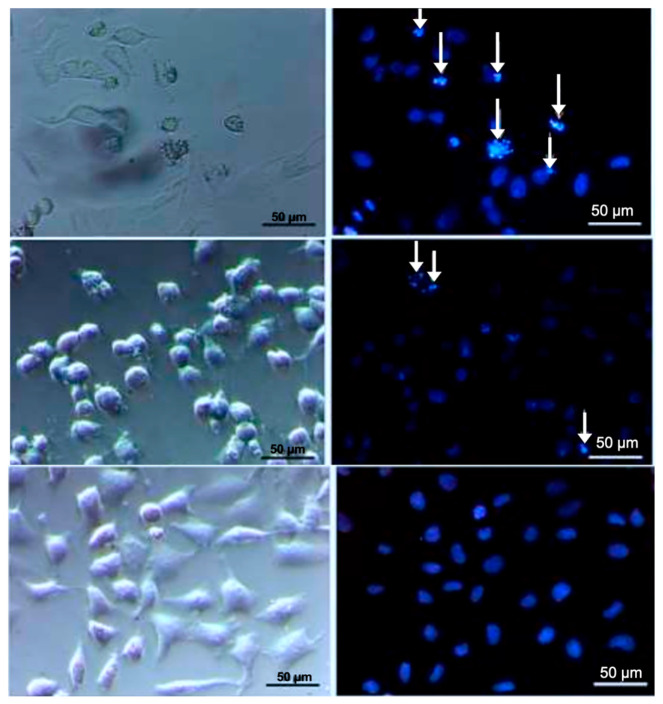
Nuclear staining of genetic material showing the formation of apoptotic bodies in HeLa cells. Phase-contrast (**left**) and fluorescence microscopy (Hoechst 33342, DAPI channel, (**right**)) images are shown for: negative control culture; culture treated with PH-Cu; and culture treated with PC-Cu, at the screening-derived working concentrations for 18 h. Intact nuclei are observed in the control culture, while arrows indicate apoptotic bodies in treated cells. Scale bar: 50 µm.

To elucidate the mechanism of action, HeLa cells were prioritized for subcellular analysis based on their observed susceptibility to both complexes. Hoechst 33342 nuclear staining following 18 h exposure at the screening-derived working concentrations revealed that both complexes induce distinct nuclear morphological aberrations specifically pyknosis and karyorrhexis, accompanied by features consistent with regulated cell death rather than disorganized necrosis (Figure 7).

These morphological observations are suggestive but not, by themselves, diagnostic of a specific death modality. These nuclear hallmarks are consistent with findings reported by Ambika et al. [57] and Paliwal et al. [48] for analogous Cu(II)–phenanthroline mixed complexes, demonstrating that the 1,10-phenanthroline unit within mono-phenanthroline scaffolds is sufficient to trigger nuclear condensation without requiring extended aromatic surfaces or polymeric stabilization.

Fluorescence microscopy analysis of the cytoskeletal architecture in PH-Cu-treated cells suggested microtubule disorganization, characterized by an apparent loss of the well-defined filamentous network observed in untreated control cells. This observation was based on a single representative field and was not quantified; imaging of the cytoskeleton was limited to PH-Cu and control conditions, and was performed in samples separate from those used for nuclear staining. Given these limitations, this finding should be regarded as preliminary evidence of cytoskeletal involvement rather than a confirmed apoptotic phenotype, and does not, on its own, establish a coordinated or simultaneous dual-target disruption mechanism within the same cells.

**Figure 8:**
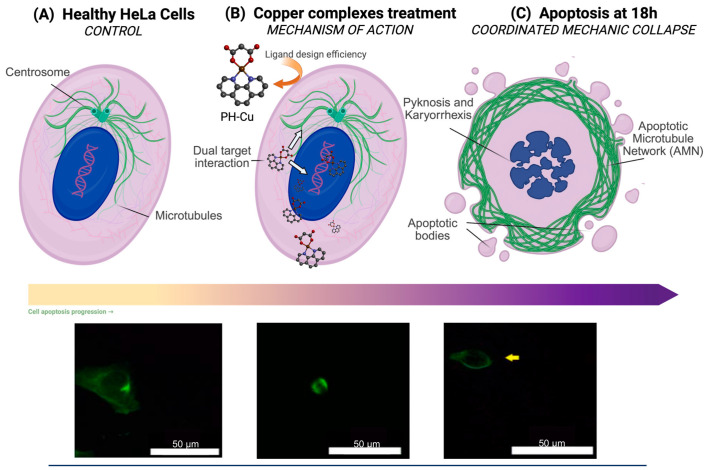
Comparative analysis of the cytoskeleton in HeLa cells through conceptual modeling and experimental validation. The top panel presents a graphical visualization of cytoskeletal disorganization as a conceptual illustration. The bottom panel displays FITC-channel fluorescence microscopy of α-tubulin staining: (**A**) A HeLa cell in G1 phase (control culture), showing homeostatic microtubule organization; (**B**) HeLa cell in metaphase (control culture), exhibiting the characteristic mitotic spindle; and (**C**) HeLa cell showing apparent microtubule disorganization following treatment with the screening-derived working concentration of PH-Cu (1.23 mg/L, 3.38 µM) for 18 h. This morphological transition is suggestive of cytoskeletal involvement but, in the absence of caspase-3 co-staining and quantification across multiple cells, does not by itself establish a confirmed apoptotic endpoint. Shown here are representative single fields; images are illustrative and were not quantified. Imaging was limited to PH-Cu and control conditions. This was created in BioRender. Garibaldi, A. (2026) https://BioRender.com/ms18807.

Microtubule disorganization following treatment with phenanthroline-based copper complexes has similarly been reported by Rostas et al. [58] suggesting that Cu(II)–phenanthroline scaffolds may affect cytoskeletal architecture in cancer cells. Notably, PH-Cu induced the nuclear apoptotic hallmarks described above within 18 h of exposure, compared to the 24–48 h timeframes reported for structurally related copper complexes by Rostas et al. [58], and Bao et al. [59] suggesting a more efficient induction of the apoptotic cascade (Figure 8). While Mukherjee et al. [60] reported direct microtubule-disrupting activity for a copper–plumbagin complex (IC_50_ = 0.85 µM in HeLa cells) through binding at the colchicine site and inhibition of tubulin polymerization, the precise molecular mechanism by which PH-Cu may interact with the microtubule network remains to be established. Confirming a defined apoptotic microtubule phenotype in this system, and extending the analysis to PC-Cu, would require higher-resolution imaging across a larger number of cells per condition and co-staining with an apoptotic marker such as cleaved caspase-3 or DAPI, which were not performed in the present study and remain important directions for future work.

It is important to note that the current study focuses on a panel of malignant cell lines to evaluate antiproliferative potency. As no non-malignant control cell line was included in this exploratory phase, the Selectivity Index (SI) could not be calculated. Consequently, while our data suggest potent and differential antiproliferative effects among the cancer models tested, these findings cannot yet be extrapolated to establish a therapeutic window relative to healthy tissue. Future studies incorporating non-transformed cell models are required to formally validate the tumor-selective profile of these complexes.

### 3.6. Transcriptional Profiling of Key Apoptotic and Regulatory Genes in HeLa Cells

**Figure 9:**
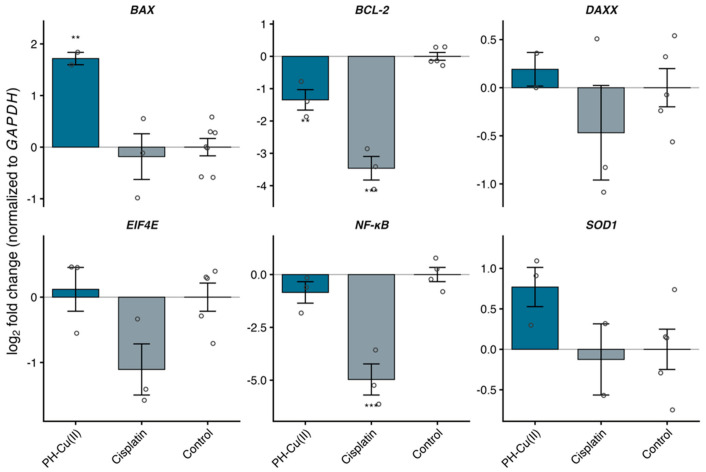
Relative mRNA expression (log_2_ fold change normalized to *GAPDH*) of key apoptotic and stress-response genes in HeLa cells after 12 h of exposure to PH-Cu(II) or cisplatin, relative to the untreated control. Bars represent mean ± SEM of *n* = 3 independent biological replicates per group; open circles show individual replicates. Asterisks denote significance vs control (one-way ANOVA followed by Dunnett post-hoc test: ** *p* < 0.01, *** *p* < 0.001).

The HeLa human cervical adenocarcinoma cell line was selected for mechanistic investigation based on its highest sensitivity among the screened panel, as evidenced by the lowest IC_50_ values recorded for both complexes. Transcriptional profiling via RT-qPCR at 12 h post-exposure revealed distinct gene expression signatures (Figure 9), with PH-Cu and PC-Cu inducing markedly different regulatory responses.

For PC-Cu(II), the reference gene *GAPDH* showed a marked amplification shift (≈6–8 cycles higher Cq than the untreated control across plates), whereas the target-gene Cq values shifted only ~1–2 cycles. Because the 2^−ΔΔCt^ method requires a stable reference, this reference-gene shift invalidates normalization for PC-Cu, and the resulting apparent fold-changes are a mathematical artifact of the collapsed denominator rather than genuine transcriptional induction. The PC-Cu data are therefore not quantified here and are presented descriptively, using raw Cq values, in Supplementary Figure S2. This shift is compatible with either a global transcriptional shutdown or compromised RNA template integrity; the present data, based on a fluorometric integrity check (Qubit RNA IQ) without an electrophoretic RIN assessment, cannot distinguish between these possibilities. Quantitative transcriptional analysis was consequently restricted to PH-Cu and cisplatin. For PH-Cu, *SOD1* increased moderately (log_2_FC = 0.77; 1.71-fold). As *SOD1* transcript levels are not a direct measure of reactive oxygen species, this change is interpreted only as a possible transcriptional indication of redox involvement and not as evidence of oxidative stress per se.

PH-Cu modulated the *BAX*/*BCL2* axis in a targeted manner, with a 3.20-fold increase in BAX (log_2_FC = 1.68) accompanied by a reduction in *BCL2* to 0.39-fold of the control value (log_2_FC ≈ −1.36) suggesting a pro-apoptotic transcriptional ratio compatible with engagement of the intrinsic apoptotic pathway. We note, however, that a transcript-level *BAX*↑/*BCL2*↓ pattern does not by itself discriminate apoptosis from other copper-associated regulated cell-death modalities, such as cuproptosis, paraptosis, or ferroptosis, which Cu(II)–phenanthroline systems are known to engage. Confirmation of the specific death pathway requires protein-level and functional validation (e.g., Annexin V/PI flow cytometry, caspase-3/7 activity, cleaved-PARP/caspase-3 immunoblot, JC-1 mitochondrial membrane potential, or cytochrome-c release), which was beyond the scope of the present study and is the subject of ongoing work. This targeted modulation of the mitochondrial rheostat aligns with findings reported by Al-Serwi et al. [61] for Cu(II) coordination compounds, where upregulation of BAX and downregulation of *BCL2* were identified as key mediators of programmed cell death in cancer cell models. Furthermore, the pro-apoptotic transcriptional signature induced by PH-Cu, characterized by *BAX* upregulation (3.20-fold) and *BCL2* downregulation (0.39-fold), is consistent with the gene expression pattern reported by Mohammadizadeh et al. [62] for the analogous Cu(II)–phenanthroline complex [Cu(L)(phen)], where a significant increase in *BAX* expression and a significant decrease in BCL2 expression were observed in cancer cells following RT-PCR analysis at their respective IC_50_ concentrations. In that study, the *BAX*/*BCL2* ratio increased 22-fold in MCF-7 cancer cells after 24 h of treatment, while only a 1.4-fold increase was observed in non-cancerous L929 cells, demonstrating preferential activation of the intrinsic apoptotic pathway in malignant cells. Although the present study evaluates transcriptional changes at an earlier timepoint (12h) and in a different cancer cell model (HeLa), the directional pattern of *BAX*/*BCL2* modulation observed for PH-Cu mirrors these findings, further supporting the activation of the intrinsic apoptotic pathway by Cu(II)–phenanthroline coordination compounds.

Comparison with cisplatin revealed a mechanistically distinct transcriptional profile. Cisplatin exhibited a predominantly repressive signature characterized by near-complete suppression of *NFKB1* (0.03-fold) and *BCL2* (0.09-fold), consistent with its well-established mechanism of DNA crosslinking and passive transcriptional shutdown. In contrast, the relative stability of eIF4E expression in PH-Cu-treated cells (1.09-fold) suggests that its cytotoxic mechanism does not rely on global translational inhibition, but rather on a coordinated regulatory transition toward programmed cell death. Taken together, the transcriptional data support a model in which PH-Cu activates the intrinsic apoptotic pathway through selective modulation of pro- and anti-apoptotic effectors, whereas PC-Cu produced a global reference-gene Cq shift that precluded quantitative transcriptional analysis (Figure 9).

### 3.7. DFT Optimized Structures of the Copper(II) Complexes

The ground-state geometries of PH-Cu and PC-Cu were optimized at the B3LYP/def2-TZVP/SVP level of theory in the gas phase. These calculations were used to validate the gas-phase coordination geometry of the anhydrous complexes against crystallographic data for analogous systems; they were not intended to predict solution-phase structure or behavior, since implicit solvation (e.g., CPCM/SMD water) was not applied and the anhydrous, singly-coordinated species was the only one modeled. The absence of imaginary frequencies in the vibrational analysis confirmed that all stationary points correspond to true local minima on the potential energy surface. Figure 9 illustrates the three-dimensional arrangements of the complexes, where the Cu(II) center is coordinated in a distorted square-pyramidal environment. This coordination sphere is formed by two nitrogen atoms from the phenanthroline-based ligand and two oxygen atoms from the secondary ligand. The B3LYP/def2-TZVP/SVP level of theory employed in this study for the geometry optimization of Cu(II) complexes has been previously validated for structurally related systems. Specifically, Khan et al. [63] employed B3LYP/def2-TZVP/def2-SVP in ORCA for the geometry optimization of heteroleptic Cu(I) complexes containing carboxylate ligands, reporting excellent agreement between calculated and experimental bond lengths and angles. Similarly, B3LYP/def2-TZVP has been applied to Cu(II) carboxylate complexes in distorted square pyramidal geometry by Kansiz et al. [64], and to Cu–phenanthroline systems by Conradie et al. [65], supporting the reliability of this functional and basis set combination for describing the coordination environment of copper(II) mixed-ligand complexes.

**Figure 10:**
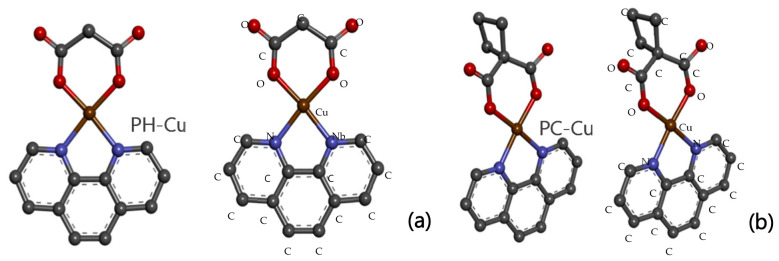
DFT-optimized ground-state geometries of PH-Cu and PC-Cu calculated at the B3LYP/def2-TZVP/SVP level. Ball-and-stick models highlight the distorted square-pyramidal coordination environment around the Cu(II) center. (**a**,**b**) Atom colors: Cu (brown), N (blue), O (red), and C (dark gray).

Optimized ground-state geometries for PC-Cu and PH-Cu (Figure 10, Table 3) were obtained at the B3LYP/def2-TZVP/SVP level, with vibrational analysis confirming all stationary points as local minima. Complex PH-Cu adopts a trans-coordinated configuration, closely resembling the centrosymmetric motif of the reference Cu1 [51], with trans axial N–Cu–O angles of ≈174°. Conversely, PC-Cu exhibits an isomeric shift to a cis-coordinated arrangement (≈92°), likely driven by the steric bulk of its cyclic backbone destabilizing the trans-form.

Calculated Cu–O (1.91–1.92 Å) and Cu–N (2.00–2.03 Å) distances show high fidelity to experimental data (1.897 Å and 2.001 Å [51]). Finally, the N–Cu–N bite angles (82.1–83.1°) reflect the intrinsic distortion of the square-pyramidal coordination sphere imposed by the phenanthroline framework, which defines the electronic environment for subsequent biological interactions.

The optimized bond lengths and angles for both complexes are in close agreement with crystallographic data reported for structurally related Cu(II)–phenanthroline systems. The calculated Cu–N distances of 2.005–2.028 Å compare favorably with the experimental value of 2.0013 Å reported by Babić et al. [51] for the crystallographically characterized reference complex Cu1, and with the range of 2.005–2.029 Å documented by Yang et al. [66] for [Cu(phen)(chloroacetate)_2_(H_2_O)]. Similarly, the calculated Cu–O distances of 1.910–1.922 Å are consistent with the experimental value of 1.8976 Å reported by Babić et al. [51] and with the range of 1.943–1.966 Å reported by Yang et al. [66] for analogous carboxylate-coordinated systems. The N–Cu–N bite angles of 82.13° (PH-Cu) and 83.09° (PC-Cu) are characteristic of the geometric constraint imposed by the 1,10-phenanthroline chelate framework, consistent with the O–Cu–N angles of 88.83–91.17° reported by Babić et al. [51] for the square-planar Cu1 reference structure. Additionally, the equatorial Cu–N distances of 2.042–2.064 Å and the Jahn-Teller elongated axial Cu–N bonds of 2.255–2.270 Å documented by de Souza Junior et al. [67] for Cu(II)–phenanthroline phases further confirm that the predominantly square-pyramidal geometry modeled for PH-Cu and PC-Cu is characteristic of mono-phenanthroline Cu(II) complexes with bidentate carboxylate co-ligands. Collectively, the close correspondence between the DFT-optimized parameters and the experimental crystallographic data from these independent structures supports the reliability of the B3LYP/def2-TZVP/SVP level of theory for describing the coordination environment of these Cu(II) mixed-ligand complexes. Two limitations of these gas-phase calculations should be noted. First, no implicit solvation model (CPCM/SMD) was applied, so the optimized geometries describe the isolated molecule rather than the solvated species. Second, only the anhydrous complex was optimized; the axial aqua ligand suggested by our own ESI-MS data ([Cu(phen)(mal)(H_2_O)], *m*/*z* 363.6) was not included, and its coordination could shift the geometry toward a square-pyramidal arrangement in solution. Accordingly, the close agreement between the calculated and crystallographic bond lengths constitutes a post-hoc validation of the gas-phase coordination geometry and should not be read as predictive of solution-phase structure.

### 3.8. Machine Learning-Based Target Identification and Mechanistic Mapping

To identify the potential therapeutic targets of the coordination complexes, an in silico interactome mapping was performed using a dual-server consensus. The following target predictions are hypothesis-generating and not experimentally validated; they are presented to prioritize candidates for future biochemical testing rather than as evidence of engagement. We further note that SuperPred and TargetNet are trained on organic small molecules, and their applicability to a paramagnetic d^9^ Cu(II) center with a labile coordination sphere is unvalidated; predictions should therefore be read as exploratory. Among the top-ranked candidates (probability > 75%), we focus on the three most defensible: PRKCG, MDM2, and the NF-κB/RELA axis.

Specifically, *PRKCG* and *MDM2* emerged as high-confidence candidates, exhibiting probability/precision scores of 99%/84% and 99%/77%, respectively, though these scores reflect statistical associations derived from structural similarity to known bioactive molecules rather than experimentally confirmed binding events.

The prediction of PRKCG as a high-confidence target is supported by the findings of Vitaliti et al. [68], who elucidated the role of copper as a dynamic modulator of intracellular signaling through the orchestration of protein kinase activity, suggesting that Cu(II) coordination complexes may preferentially engage metal-binding domains within kinase regulatory regions to modulate signaling cascades aberrantly upregulated during oncogenesis. Similarly, the predicted prioritization of MDM2 is consistent with the framework proposed Alfadul et al. [69], who characterized the p53-MDM2 axis as a critical regulatory locus in neoplastic progression and a pivotal focal point in the investigation of metal-based coordination complexes.

This prediction warrants caution: PRKCG (PKCγ) is predominantly expressed in central nervous system tissue, particularly the cerebellum, and its physiological relevance in cervical adenocarcinoma (HeLa) is uncertain. Its appearance here likely reflects structural similarity to kinase-targeting bioactive molecules in the training set rather than a validated HeLa-specific interaction, and we present it only as a structural-similarity-driven hypothesis requiring experimental confirmation.

The predictive mapping further suggests potential association with NFKB1 (78% prob., 96% prec.), RELA, and CASP9 (both 100% prob.). Although the computational models initially ranked BCL2A1 among the top candidates, the prototypical BCL2 was prioritized for downstream evaluation given its fundamental role in governing the mitochondrial apoptotic rheostat. The predicted involvement of the NF-κB pathway is consistent with the findings reported by McElwee et al. [70], who reported that copper exposure may catalyze NF-κB-mediated transcription, and with the mechanistic framework proposed by Persichini et al. [71], who described the potential activation of the p50/p65 dimer through metal-induced oxidative stress. These precedents suggest that PH-Cu and PC-Cu could potentially modulate pro-inflammatory gene expression, including COX-2 and TNF-α, though the extent to which this predicted interactome translates into attenuation of tumor-promoting inflammation in HeLa cells remains to be confirmed experimentally. Similarly, the high probability score obtained for CASP9 (100%) is supported by the findings of Vitomirov et al. [72], whose investigations into copper-phenanthroline complexes demonstrated the induction of apoptosis through activation of the caspase cascade involving caspases-3, 8, and 9, suggesting that the present complexes may engage a comparable apoptotic mechanism, contingent on experimental validation.

**Figure 11:**
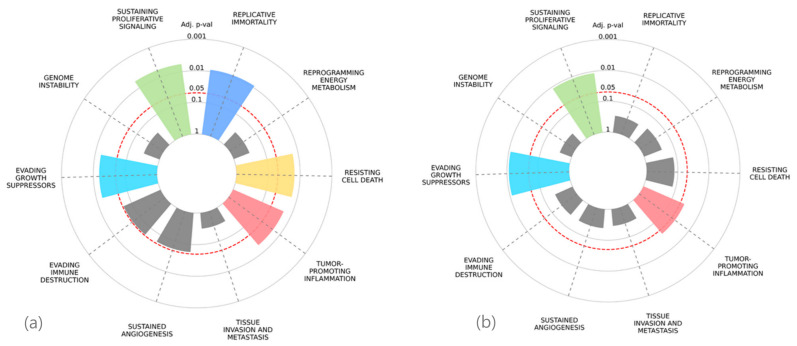
Comparative functional enrichment analysis of the predicted molecular interactomes based on the Hallmarks of Cancer. (**a**) Hallmark enrichment profile for the PH-Cu complex. (**b**) Hallmark enrichment profile for the PC-Cu complex. The red dashed line represents the statistical significance threshold (adj. *p* < 0.05) determined by a hypergeometric test. The background gene set (universe) consisted of all annotated human protein-coding genes in the platform’s database, and *p*-values were adjusted for multiple testing using the Benjamini-Hochberg False Discovery Rate (FDR) method. Colored sectors indicate biological capabilities with significant overrepresentation, while gray sectors represent non-significant associations. This figure was created with CancerHallmarks [73].

A functional enrichment analysis (Figure 11) indicated overrepresentation of cancer-hallmark categories (e.g., Sustaining Proliferative Signaling, Evading Growth Suppressors, Resisting Cell Death) in the predicted interactomes of both complexes. As these enrichments derive from the same unvalidated predictions, they are presented as exploratory hypotheses for prioritizing future targeted validation rather than as mechanistic conclusions.

Despite the predictive power of the implemented computational pipeline, several limitations inherent to the application of machine learning-based approaches to metal coordination complexes must be acknowledged. As highlighted by Lee et al. [74] conformer generation and property prediction methods developed for organic molecules are not directly applicable to coordination complexes due to their structural diversity, variable coordination geometries, and stereochemical complexity. Furthermore, Rasmussen et al. [75] demonstrated that standard SMILES representations of transition metal complexes present fundamental interoperability limitations, with approximately half of the SMILES strings available in the Cambridge Structural Database being unparseable by standard cheminformatics tools such as RDKit. Consequently, the target prediction platforms employed herein, primarily trained on organic small molecule datasets, may not fully account for the three-dimensional coordination environment, the oxidation state of the Cu(II) center, or the dynamic lability of metal–ligand bonds under physiological conditions. The predicted probability scores should therefore be interpreted as hypothesis-generating rather than conclusive, and experimental validation of the predicted targets through biochemical assays remains essential to confirm the mechanistic hypotheses proposed herein.

### 3.9. ADMET Modeling and Toxicological Divergence

In silico consensus modeling through ADMETlab 3.0 and pkCSM predicted favorable drug-likeness profiles for both complexes, characterized by superior human intestinal absorption (>92.8%), moderate aqueous solubility (logS between −2.4 and −2.7), and Caco-2 permeability values (>1.18 logP app) surpassing established efficiency thresholds. These predicted parameters are consistent with findings reported by Babić et al. [51] for structurally analogous Cu(II) systems, and contrast with the oral bioavailability reduction observed by Helaly et al. in Cu(II) Salen-type complexes upon metal complexation, suggesting that the 1,10-phenanthroline/dicarboxylate scaffold may confer a more favorable absorption profile [76]. It should be noted that ADMET platforms are primarily trained on organic small molecule datasets, and their applicability to Cu(II) coordination compounds requires experimental validation.

Regarding predicted distribution and metabolism, both complexes showed low steady-state volumes of distribution (VD ss < −0.03 logL/kg) and consistently low BBB permeability (logPS < −2.38) across all platforms, a pharmacologically desirable feature for peripherally acting antineoplastic agents that would reduce the risk of secondary neurotoxicity. Metabolic profiling suggested CYP3A4 substrate behavior with negligible inhibitory activity across major CYP450 isoforms, consistent with the enzymatic stability reported by Helaly et al. [76] for analogous systems, and implying a potentially reduced risk of drug–drug interactions. Toxicological modeling predicted hepatotoxicity and ARE-mediated (antioxidant–response–element) activity; as these are computational predictions and no direct ROS measurement was performed, they are presented as hypotheses rather than confirmation of an oxidative-stress mechanism.

Deep learning evaluation via Tox21 suggested a mechanistic divergence between the two complexes: PC-Cu exhibited a predicted capacity to disrupt mitochondrial membrane potential and activate AAA-ATPases, effects markedly attenuated in PH-Cu. This computational distinction is consistent with the differential transcriptional response observed in the RT-qPCR profiling (Section 3.6), and suggests a predicted divergence in mitochondrial/bioenergetic effects between the two complexes; this remains a computational prediction that requires direct experimental confirmation (e.g., ROS and mitochondrial assays). As with all in silico predictions, these findings should be interpreted as mechanistic hypotheses pending confirmation through experimental pharmacokinetic and metabolic stability assays.

### 3.10. Molecular Docking Analysis of Apoptosis-Related and Structural Targets

**Figure 12:**
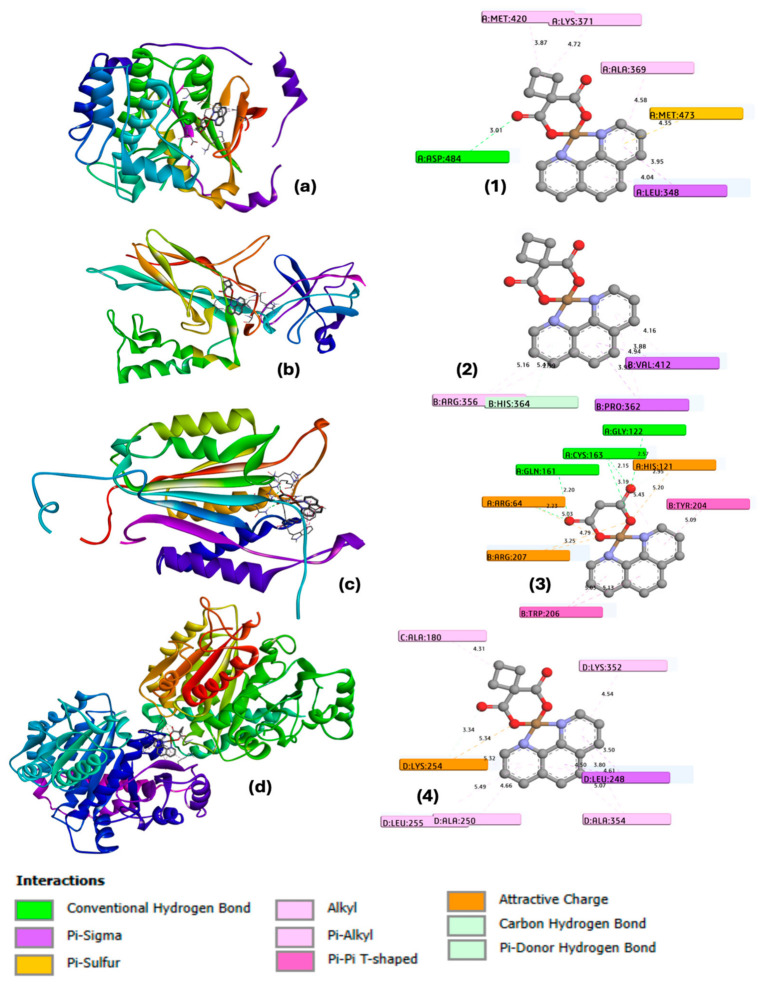
Molecular docking analysis of the coordination complexes with their primary biological targets. The left column displays the three-dimensional (3D) binding poses within the catalytic or regulatory pockets of the receptors, while the right column shows the corresponding two-dimensional (2D) interaction maps. (**a**) PC-Cu (1) interaction with PRKCG (PDB ID: 2I0E); (**b**) PC-Cu (2) docking within the RELA subunit (PDB ID: 1VKX); (**c**) PH-Cu (3) binding at the active site of Caspase-3 (PDB ID: 3KJF); and (**d**) PC-Cu (4) occupying the colchicine-binding site of α/β-tubulin(PDB ID: 1SA0).

The binding energies (ΔG) and theoretical inhibition constants (Ki) for complexes PH-Cu and PC-Cu against critical apoptotic and structural targets are summarized in Table 4. The docking simulations (Figure 12) suggest a consistent trend in which PC-Cu exhibits a higher binding rank than PH-Cu across the majority of evaluated proteins, with the strongest predicted binding for PRKCG and α/β-tubulin (relative ranking; Table 4). These ranks position PRKCG and tubulin as potential primary scaffolds for the action of PC-Cu, though experimental validation is required.

Within the mitochondrial apoptotic pathway, PC-Cu displayed a significantly stronger predicted affinity for *BAX* (ΔG = −8.9kcal/mol [−37.2 kJ mol^−1^]) compared to PH-Cu (ΔG = −6.7 kcal/mol [−28.0]), suggesting a possible molecular basis for its enhanced cytotoxic potential observed in vitro. Conversely, both compounds exhibited comparable predicted affinities for Caspase-3 ΔG of −8.9 [−37.2 kJmol^−1^] and −8.6 kcal/mol [−36.0 kJ mol^−1^], respectively, indicating a potentially shared capacity to trigger the execution phase of apoptosis. Interaction fingerprint analysis indicates that predicted binding sites are predominantly stabilized by a network of hydrophobic residues, including ALA, LEU, and VAL [77], complemented by π-mediated interactions with aromatic residues such as PHE, TYR, and TRP [78]. Specifically, the superior affinity of PC-Cu toward PRKCG may be attributed to a broader interaction surface involving specialized residues such as MET420 and THR404, which are not consistently engaged by PH-Cu.

A notable internal inconsistency must be acknowledged: although the ML target-prediction step ranked CASP9 with high probability (100%), docking returned its weakest score for this target. This divergence between similarity-based prediction and structure-based docking, for a compound class for which neither method is validated, means CASP9 cannot be regarded as a supported target; we present it only as an example of the limited concordance between the two computational layers, requiring experimental adjudication.

This docking model suggests that PC-Cu could establish a primary polar anchor through conventional hydrogen bond with THR484, further complemented by π-Sigma-type interactions with LEU348, π-Sulfur contacts with MET473, and Alkyl interactions with MET420 and LYS371. These features, if validated experimentally, would suggest PRKCG as a relevant target for PC-Cu-mediated disruption of survival signaling. This is consistent with the findings of Mashozhera et al. [79], who reported an 8.81-fold upregulation of PRKCG in response to potent anticancer agents, and with the multi-omics analysis of Zhao et al. [80] which designates PRKCG as a key node in complex therapeutic interventions.

Regarding the NF-κB pathway, the docking results suggest a potentially relevant interaction between both complexes and RELA(p65) subunit. The predicted binding mode of PC-Cu at the p65 interface involves a π-donor hydrogen bond with HIS364 as a primary electronic anchor, supplemented by π-Sigma contacts with VAL412 and PRO362, and hydrophobic π-Alkyl contributions with ARG356. These computational results are consistent with the precedent reported by Caetano-Silva et al. [81] who demonstrated that copper-coordinated systems may exert inhibitory effects on the NF-κB pathway through direct binding to p65 (ΔG = −8.8 kcal/mol), potentially reducing its nuclear translocation. While the present docking results align with this mechanistic framework, the extent to which PH-Cu and PC-Cu may suppress NF-κB-dependent pro-survival signaling in HeLa cells remains to be confirmed through reporter assays or nuclear fractionation studies.

For Caspase-3, the predicted binding configuration of PH-Cu involves a dense network of polar and electrostatic interactions, including conventional hydrogen bonds with GLN161, CYS163, and GLY122, attractive charge interactions and salt bridges with ARG64, ARG207, and HIS121, and π-π T-shaped contacts with TYR204 and TRP206. This interaction profile suggests a potentially targeted engagement with the enzyme’s executioner active site, and compares favorably with the binding architecture reported by Kashaaw et al. [82] for 1,2,4-oxadiazole derivatives, where ASN273 was identified as the primary stabilizing residue. The distinct residue engagement pattern observed for PH-Cu may reflect the particular electronic properties of the phenanthroline scaffold, though direct comparison requires experimental binding data.

Finally, the predicted binding mode of PC-Cu at the α/β-tubulin interface involves a central attractive charge interaction with LYS254, complemented by a carbon hydrogen bond with the same residue, alkyl contacts with ALA180, and an extended network of π-Alkyl interactions with LYS352, ALA354, LEU248, ALA250, and LEU255. This binding configuration is comparable to that described by Mukherjee et al. [60] for the Cu-PLN complex at the colchicine-binding site (ΔG = −9.8 kcal/mol), and suggests that PC-Cu (ΔG = −9.9 kcal/mol) may adopt a similar strategic positioning near the T7-loop. If confirmed experimentally, interactions with residues such as GLN247 and THR353 could potentially hinder the conformational flexibility required for tubulin polymerization, consistent with the microtubule reorganization observed in fluorescence microscopy of HeLa cells treated with both complexes. The potential for multi-target engagement is further supported by the findings of Milunovic et al. [83] in thiosemicarbazone-based copper systems, which exhibited dual functionality through simultaneous tubulin inhibition and ribonucleotide reductase disruption, suggesting that copper-based frameworks may bypass conventional multidrug resistance mechanisms through this multi-modal strategy.

It should be noted that the AutoDock Vina scoring function is parameterized for organic ligands and does not explicitly treat copper coordination chemistry; the ΔG values reported here should therefore be interpreted as relative, hypothesis-generating rankings rather than quantitative affinities, and require experimental validation (e.g., enzymatic inhibition or microscale thermophoresis). Moreover, these simulations assume retention of the intact coordination sphere upon target binding; in practice, Cu(II) complexes may undergo partial ligand exchange with coordinating amino-acid residues, so the predicted poses should be regarded as approximations of the bound species.

## 4. Conclusions

Two novel Cu(II)–phenanthroline coordination complexes, PH-Cu and PC-Cu, were synthesized and characterized. The obtained results from experimental structural analysis (FTIR-ATR, ESI-MS, and EPR) were consistent with the proposed distorted square-pyramidal architectures. The inactivity of all precursor compounds (IC_50_ > 1109 µM) and the comparable potency of PH-Cu, PC-Cu, and [Cu(phen)Cl_2_] support the interpretation that the [Cu(phen)] unit constitutes the primary pharmacophore, with the dicarboxylate ligands functioning as haptophores modulating the physicochemical properties of the complex.

Both complexes exhibited potent antiproliferative activity across six human cancer cell lines, with estimated IC_50_ values of 4.22 µM and 6.22 µM in HeLa cells, corresponding to 4–6-fold lower IC_50_ than cisplatin in HeLa under the present 24 h protocol, a margin that is cell-line- and assay-time-dependent and a 12-fold improvement over previously reported phenanthroline derivatives in HepG2 cells. Subcellular analysis at 18 h revealed morphological features suggestive of pyknosis, karyorrhexis, and microtubule disorganization, consistent with regulated cell death at an earlier timepoint than reported for structurally related copper complexes. RT-qPCR profiling revealed mechanistically distinct responses: PH-Cu produced a transcriptional signature consistent with intrinsic apoptotic pathway engagement (BAX +3.20-fold; BCL2 to 0.39-fold of control), pending confirmation by protein-level and functional cell-death assays. Both profiles are mechanistically distinct from the predominantly repressive transcriptional signature of cisplatin (NFKB1: 0.03-fold; BCL2: 0.09-fold).

DFT optimization yielded Cu–O (1.91–1.92 Å) and Cu–N (2.00–2.03 Å) bond lengths consistent with experimental data. Chemoinformatic target prediction and molecular docking suggested PRKCG, RELA (p65), Caspase-3, and α/β-tubulin as potential interaction candidates (strongest predicted binding ranks; see Section 3.10), while ADMET modeling predicted favorable intestinal absorption (>92.8%) and low BBB permeability (logPS < −2.38). Collectively, these results suggest that phenanthroline-based Cu(II) dicarboxylate complexes represent a promising exploratory scaffold warranting further investigation as potential anticancer agents, particularly in cisplatin-resistance cell models.

Nevertheless, several limitations must be acknowledged. The computational predictions reported herein are inherently constrained by the applicability of organic molecule-trained machine learning platforms to Cu(II) coordination compounds, as standard SMILES-based representations may not fully capture the three-dimensional coordination geometry, metal oxidation state, or metal-ligand bond lability under physiological conditions. Furthermore, solution stability data and selectivity profiles toward non-cancerous cell lines remain to be established. The molecular formula and coordination geometry proposed here rest on ESI-MS, FTIR-ATR, EPR, and DFT, without single-crystal X-ray diffraction, powder X-ray diffraction (PXRD), or elemental (CHN) analysis. While this combination is adequate to support the structural assignment for a preliminary biological screen, it does not constitute a definitive solid-state structural determination; single-crystal XRD, PXRD, and CHN are required to confirm the proposed structures and are a priority for future work. Experimental biochemical validation of the predicted molecular targets is therefore essential to consolidate the mechanistic framework proposed in this study and to define the therapeutic window of this compound family as candidates for preclinical development.

Additionally, while the exploratory MTT screening successfully established reliable estimated IC_50_ thresholds for our targeted morphological and RT-qPCR evaluations, the statistical determination of absolute IC_50_ values across multiple biological replicates remains a limitation of the current experimental design. Future high-throughput pharmacological profiling will be required to establish definitive toxicological and safety parameters.

Finally, the cell-death modality was inferred from morphological and transcriptional readouts only; protein-level and functional assays (Annexin V/PI, caspase-3/7 activity, cleaved-PARP/caspase-3, JC-1, cytochrome-c release) were not performed, and alternative copper-associated regulated cell-death pathways (cuproptosis, paraptosis, ferroptosis) cannot be excluded. Discriminating among these is a priority for future validation.

Cisplatin reference IC_50_ values were obtained under a single 24 h exposure protocol and the working stock was not independently verified spectrophotometrically; given the strong assay- and time-dependence of cisplatin potency, the relative advantage of the copper complexes should be interpreted within these defined conditions rather than as an absolute pharmacological ranking.

## Figures and Tables

**Table 1:** Main infrared (FTIR) spectroscopic signals and their corresponding vibrational modes.

Signal	Bond	Vibration	Range	Reference
A	C–H	Aromatic stretching	3000–3100	[37]
B	COO^−^	v_as_(COO^−^) asymmetric stretching	1600–1700	[38]
C	C=C/COO^−^	Aromatic stretching	1300–1400	[37]
D	C–N	Aromatic stretching	1050–1150	[37]
E	C–H	Out-of-plane bending (central ring)	800–900	[39]
F	C–C	Skeletal ring vibration	750–800	[40]
G	C–H	Out-of-plane bending (heterocycle)	700–750	[39]

**Table 2:** Estimated working IC_50_ values (µM) and 95% confidence intervals (95% CI) for the synthesized complexes and cisplatin across the evaluated cancer cell lines. Values were determined using a constrained sigmoidal four-parameter logistic (4PL) non-linear regression model.

Compound	Cell Line	Estimated IC_50_ (µM)	95% CI (µM)
**Cisplatin**	HeLa	21.93	20.03–23.83
**Cisplatin**	HCC1954	18.33	14.26–22.36
**Cisplatin**	Jurkat	9.73	8.36–11.10
**Cisplatin**	HepG2	191.13	86.48–295.75
**Cisplatin**	Caco-2	129.61	94.81–164.43
**PH-Cu**	HeLa	4.22	3.79–4.63
**PH-Cu**	HCC1954	6.59	6.16–7.03
**PH-Cu**	Jurkat	5.18	4.71–5.64
**PH-Cu**	HepG2	8.82	7.69–9.95
**PH-Cu**	Caco-2	7.17	5.75–8.56
**PH-Cu**	NCl-H69	10.27	6.80–13.74
**PC-Cu**	HeLa	6.22	5.78–6.63
**PC-Cu**	HCC1954	8.45	7.49–9.41
**PC-Cu**	Jurkat	9.30	8.06–10.52
**PC-Cu**	HepG2	15.63	14.05–17.21
**PC-Cu**	Caco-2	8.86	6.06–11.64
**PC-Cu**	NCl-H69	13.22	7.77–18.66

**Table 3:** Selected bond lengths (Å) and bond angles (°) for the DFT-optimized ground-state geometries of complexes PH-Cu and PC-Cu.

Bond Lengths (Å)	PH-Cu	PC-Cu	Bond Angles °	PH-Cu	PC-Cu
Cu1–N5	2.028	2.008	N5–Cu1–N18	82.13	83.09
Cu1–N18	2.028	2.005	O22–Cu1–O26	93.36	91.57
Cu1–O22	1.910	1.922	N5–Cu1–O26	174.36	92.84
Cu1–O26	1.910	1.919	N18–Cu1–O22	174.41	92.49

**Table 4:** Relative docking ranking of PH-Cu and PC-Cu against the evaluated targets.

	ΔG (kcal/mol) [kJ mol^−1^]		Key Interaction Residues	
Protein Target	PH-Cu	PC-Cu	PH-Cu	PC-Cu
*PRKCG*	−8.9 [−37.2]	−10 [−41.8]	LYS371, VAL356, LEU348, ALA369, ALA483, VAL423	MET420, LYS371, THR404, ALA369, VAL423, MET473, LEU348, VAL356, ALA483
*BAX*	−6.7 [−28.0]	−8.9 [−37.2]	GLU129, LEU130, ARG139, ALA142	LEU108, PHE97, PHE105, LEU130, ALA104
*BCL2*	−7.1 [−29.7]	−7.9 [−33.1]	TYR67, ALA108, LEU96	LEU96, ALA108, TYR67
*MDM2*	−7.7 [−32.2]	−8.4 [−35.2]	SER17, GLY16, LEU54, ILE99, VAL93	SER17, LYS51, GLY16, LEU54, ILE99, VAL93
*CASP9*	−5.7 [−23.9]	−4.1 [−17.2]	PHE267, ASN268, ASN265, LYS280, THR337, ASP340	PRO338, ILE341, ASN265
*NFKB1*	−7.8 [−32.6]	−7.5 [−31.4]	GLN119, ARG41, VAL91, SER42	ALA129, LYS37, CYS38, LYS122, ASP125
*RELA*	−8.6 [−36.0]	−9.1 [−38.1]	ARG356, HIS364, PRO362, VAL412	ARG356, HIS364, PRO362, VAL412
*CASP3*	−8.9 [−37.2]	−8.6 [−36.0]	TRP206, TYR204, CYS163, HIS121, GLN161, ARG64, ARG207	TRP206, ARG207, CYS163, GLY122, HIS121
α/β Tubulin	−9.0 [−37.8]	−9.9 [−41.4]	ALA250, LEU255, LEU248, ALA354, LYS352, LYS254	ALA180, LYS254, LEU255, ALA250, LEU248, ALA354, LYS352

ΔG 1. kcal/mol = 4.184 kJ mol^−1^.

## Data Availability

The original contributions presented in this study are included in the article and Supplementary Materials.

## Supplementary Materials

The following supporting information can be downloaded at https://www.mdpi.com/article/10.3390/biomedicines14071625/s1, Table S1: Primer and probe sequences utilized for RT-qPCR analysis, including expected amplicon sizes. Table S2: Selected crystal parameters of PH-Cu obtained by SC-XRD. Table S3: Selected bond distances (Å) for PH-Cu obtained by SC-XRD. Figure S1: Single-crystal X-ray diffraction pattern of PH-Cu. Table S4: Exploratory dose-finding screening (MTT assay) of individual precursors, the dicarboxylate-free reference compound PCl_2_-Cu, and the copper complexes in HeLa cells. Figure S2: Raw quantification cycle (Cq) values illustrating reference-gene drop-out in PC-Cu treated Hla cells.
