# Supplementary material for: Experimental and Computational Insights into the Apoptotic Potential of New Phenanthroline-Based Copper(II) Complexes: From Spectroscopic Characterization and In Vitro Cytotoxicity to In Silico Target Identification
Source: Biomedicines. 2026 Jul 20;14(7):1625. doi: 10.3390/biomedicines14071625 (PMC13406101; doi:10.3390/biomedicines14071625)
Supplement: Supplementary file 1 [file biomedicines-14-01625-s001.zip › biomedicines-4388087-supplementary.pdf]

Supplementary Table S1. Primer and probe sequences utilized for RT-qPCR analysis, including expected amplicon sizes. All probes utilized a FAM reporter and ZEN/IABk double-quenching.

Target Gene	Primer / Probe Sequence (5' → 3')	Amplicon Size (bp)
**GAPDH**	**F:** GAA GGT GAA GGT CGG AGT C<br><br>**R:** GAA GAT GGT GAT GGG ATT TC<br><br>**Probe:** CAA GCT TCC CGT TCT CAG CC	226
**eIF4E**	**F:** CTA AGA TGG CGA CTG TCG AA<br><br>**R:** AGG TTT GCT TGC CAA GTT TT<br><br>**Probe:** ATC CCC CGA CTA CAG AAG AGG	184
**NFKB1**	**F:** TAT ACT TCA TGT GAC AAA G<br><br>**R:** CAG GGT GCA CCA AGA GTC C<br><br>**Probe:** AAG CAC GAA TGA CAG AGG CGT	101
**BCL2**	**F:** TTG GCC CCC GTT GCT T<br><br>**R:** CGG TTA TCG TAC CCC GTT CTC<br><br>**Probe:** AGC GTG CGC CAT CCT TCC CAG	65
**BAX**	**F:** TCC CCC CGA GAG GTC TTT T<br><br>**R:** CGG CCC CAG TTG AAG TTG<br><br>**Probe:** TCA GAA AAC ATG TCA GCT GCC ACT CGG	68
**DAXX**	**F:** CAA AAG TGA GGA GGG CGA GAG<br><br>**R:** AGG CTG TAC CCC ATC CAC ACC<br><br>**Probe:** AAG CCT CCT TGG ATT CTG GT	120
**SOD1**	**F:** AGG TGA TTG CTC TGC TGC TT<br><br>**R:** ACA GAG CAC AAC CTC GAC AG<br><br>**Probe:** TTA TGA AAG GGC CTC CTG TGC	130

Supplementary Table S2. Selected crystal parameters of PH-Cu obtained by SC-XRD

Parameter	Value
**Crystal system**	**Triclinic**
a (Å)	11.5591
b (Å)	11.7450
c (Å)	12.5081
α (°)	92.18
β (°)	105.46
γ (°)	109.18
V ($Å^3$)	1531.1

Supplementary Table S3. Selected bond distances (Å) for PH-Cu obtained by SC-XRD

Bond	Distance (Å)	Bond	Distance (Å)
Cu1 – O2	1.9061	Cu2 – O3	1.9114
Cu1 – O1	1.9270	Cu2 – O7	1.9246
Cu1 – N4	1.9890	Cu2 – N1	2.0170
Cu1 – N3	2.0298	Cu2 – N2	2.0260
Cu1 – O9	2.2930	Cu2 – O10	2.2643

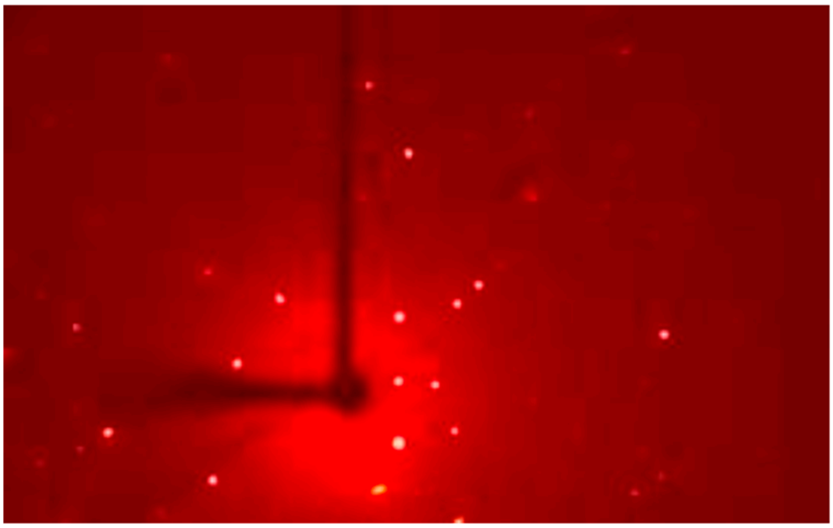

Supplementary Figure S1. Single-crystal X-ray diffraction pattern of PH-Cu. The diffraction image shows well-defined Bragg reflections distributed across the detector, consistent with a high-quality single-crystal specimen. The dark vertical and horizontal lines correspond to the beamstop used to protect the detector from the primary beam. This image confirms the crystalline nature of the synthesized PH-Cu complex and supports the crystal parameter data reported in Supplementary Table S2 and S3.

Supplementary Table S4. Exploratory dose-finding screening (MTT assay) of individual precursors, the dicarboxylate-free reference compound PCl_2-Cu, and the copper complexes in HeLa cells.

Compound	(mg/L)	μM
1,10-Phenanthroline (free ligand)	>200[a]	>1110[a]
Malonic acid (free ligand)	>200[a]	>1961[a]
Cyclobutane-1,1-dicarboxylic acid (free ligand)	>200[a]	>1407[a]
$CuCl_2$	>200[a]	>1487[a]
[Cu(phen)Cl_2] (PCl_2-Cu, dicarboxylate-free reference intermediate)	1.45	4.61

[a] *These compounds did not reach 50% growth inhibition within the maximum concentration tested (200 mg/L); values are reported as the upper limit of the tested range.*

These values correspond to the initial exploratory dose-finding screening (linear regression analysis) and are distinct from the refined IC_{50} values for PH-Cu and PC-Cu reported in Table 2, which were subsequently recalculated using a constrained 4PL non-linear regression model. Nevertheless, this screening step provides important comparative evidence: all individual precursors were essentially inactive under the tested conditions, while PCl_2-Cu, which lacks the dicarboxylate co-ligand, displayed screening activity comparable to that of PH-Cu and PC-Cu. This indicates that the [Cu(phen)] coordination unit constitutes the primary pharmacophore responsible for the antiproliferative activity, while the anionic co-ligands (chloride or dicarboxylate) function as haptophores modulating the physicochemical properties of the complex.

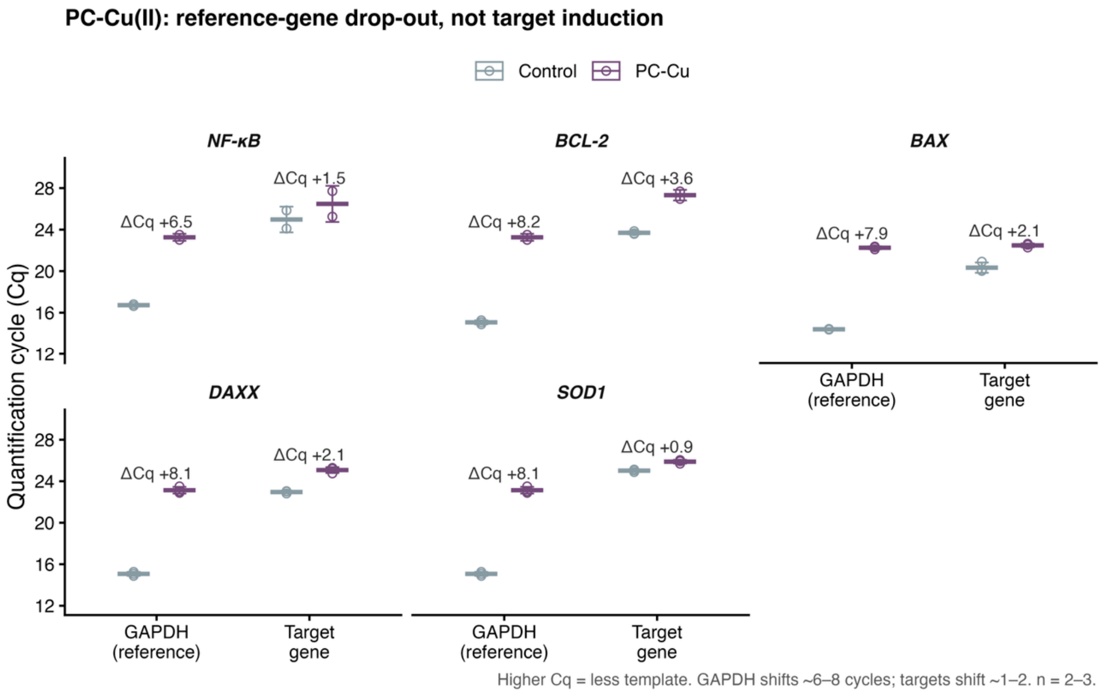

Supplementary Figure S2. Raw quantification cycle (Cq) values illustrating reference-gene drop-out in PC-Cu-treated HeLa cells. Cq values for the reference gene *GAPDH* and target genes (*NF-κB*, *BCL-2*, *BAX*, *DAXX*, *SOD1*) are shown for untreated control and PC-Cu-treated cultures. GAPDH Cq values shifted by approximately 6–8 cycles relative to control, while target-gene Cq values shifted by only 1–2 cycles, indicating reference-gene instability rather than genuine target-gene induction. Higher Cq values indicate lower template abundance. Data are presented as individual replicates with mean ± range (n = 2–3); ΔCq values relative to control are indicated above each gene pair.
